# Skeletal and Extraskeletal Actions of Vitamin D: Current Evidence and Outstanding Questions

**DOI:** 10.1210/er.2018-00126

**Published:** 2018-10-12

**Authors:** Roger Bouillon, Claudio Marcocci, Geert Carmeliet, Daniel Bikle, John H White, Bess Dawson-Hughes, Paul Lips, Craig F Munns, Marise Lazaretti-Castro, Andrea Giustina, John Bilezikian

**Affiliations:** 1 Laboratory of Clinical and Experimental Endocrinology, Department of Chronic Diseases, Metabolism and Ageing, KU Leuven, Belgium; 2 Department of Clinical and Experimental Medicine, University of Pisa, Pisa, Italy; 3 Veterans Affairs Medical Center and University of California San Francisco, San Francisco, California; 4 Department of Physiology, McGill University, Montreal, Quebec, Canada; 5 Jean Mayer USDA Human Nutrition Research Center on Aging at Tufts University, Boston, Massachusetts; 6 Department of Internal Medicine, Endocrine Section, VU University Medical Center, HV Amsterdam, Netherlands; 7 Children’s Hospital at Westmead, Sydney, New South Wales, Australia; 8 Sydney Medical School, University of Sydney, Sydney, New South Wales, Australia; 9 Division of Endocrinology, Escola Paulista de Medicina, Universidade Federal de São Paulo, São Paulo, Brazil; 10 Chair of Endocrinology, Vita-Salute San Raffaele University, Milan, Italy; 11 Department of Endocrinology, Columbia University College of Physicians and Surgeons, New York, New York

## Abstract

The etiology of endemic rickets was discovered a century ago. Vitamin D is the precursor of 25-hydroxyvitamin D and other metabolites, including 1,25(OH)_2_D, the ligand for the vitamin D receptor (VDR). The effects of the vitamin D endocrine system on bone and its growth plate are primarily indirect and mediated by its effect on intestinal calcium transport and serum calcium and phosphate homeostasis. Rickets and osteomalacia can be prevented by daily supplements of 400 IU of vitamin D. Vitamin D deficiency (serum 25-hydroxyvitamin D <50 nmol/L) accelerates bone turnover, bone loss, and osteoporotic fractures. These risks can be reduced by 800 IU of vitamin D together with an appropriate calcium intake, given to institutionalized or vitamin D–deficient elderly subjects. VDR and vitamin D metabolic enzymes are widely expressed. Numerous genetic, molecular, cellular, and animal studies strongly suggest that vitamin D signaling has many extraskeletal effects. These include regulation of cell proliferation, immune and muscle function, skin differentiation, and reproduction, as well as vascular and metabolic properties. From observational studies in human subjects, poor vitamin D status is associated with nearly all diseases predicted by these extraskeletal actions. Results of randomized controlled trials and Mendelian randomization studies are supportive of vitamin D supplementation in reducing the incidence of some diseases, but, globally, conclusions are mixed. These findings point to a need for continued ongoing and future basic and clinical studies to better define whether vitamin D status can be optimized to improve many aspects of human health. Vitamin D deficiency enhances the risk of osteoporotic fractures and is associated with many diseases. We review what is established and what is plausible regarding the health effects of vitamin D.

**Essential Points**

- Vitamin D prevents and cures nutritional rickets but implementation of an adequate prevention strategy is still problematic in many countries or for subgroups of the world population
- The near universal distribution of the vitamin D receptor and vitamin D metabolizing enzymes CYP24A1 and CYP27B1, along with the large number of genes under direct control of 1,25(OH)_2_D, all argue for a wide diversity of actions of the vitamin D endocrine system
- Most animal data are in line with human data on the role of vitamin D in helping to regulate calcium and bone metabolism
- Many observational studies link a poor vitamin D status to major human diseases
- About 38 Mendelian randomization studies have addressed, in the past several years, a link between genetically lower 25-hydroxyvitamin D concentration and skeletal or extraskeletal health outcomes; the best documented link so far is found for multiple sclerosis
- Intervention studies for extraskeletal health effects are so far inconclusive, but the results of several ongoing randomized clinical trials may help to delineate these effects more clearly

A causal role of vitamin D for bone health is well established, as vitamin D deficiency is the cause of most cases of rickets and osteomalacia. Vitamin D also plays a major role in the pathogenesis of renal osteodystrophy, and its deficiency can accelerate bone loss and osteoporosis of the elderly. Preclinical data suggest that severe vitamin D deficiency may have extraskeletal effects. Many observational studies also link a poor vitamin D status to a wide variety of extraskeletal diseases. However, cause and effect have not been confirmed, and therefore the optimal vitamin D intake or optimal levels of the major circulating metabolite 25-hydroxyvitamin D (25OHD) to achieve clinically detectable nonskeletal effects are not known.

The origin, transport, metabolism, and action of vitamin D have many similarities with those of iodide/thyroid hormones. Therefore, we compare both endocrine systems in Table 1. Vitamin D is either of dietary origin or can be synthesized in the skin under the influence of UV-B light. The dietary intake of vitamin D (mostly D_3_ and minimal amounts of D_2_) is usually low. Similarly, iodide intake is variable and depends on geography and dietary habits. For both systems, a complex system allows first the storage of precursor molecules in tissues (Table 1), followed by secretion of a prohormone in the blood where it is bound to specific transport proteins. Thereafter, the hormone precursors (25OHD and T_4_, respectively) can be activated into the hormone [1*α*,25-dihydroxyvitamin D (1,25(OH)D) and T_3_, respectively] or inactived into other metabolites [initially mainly 24R,25(OH)_2_D and reverse T_3_, respectively, but followed later by many other metabolites]. The active hormones have a high affinity for their respective nuclear receptors [vitamin D receptor (VDR) and thyroid hormone receptor] but much lower affinity for their serum transport proteins, thereby favoring the selective nuclear uptake of the hormones whereas the precursor preferentially remains in the bloodstream. The essential aspects of vitamin D metabolism and action are presented in Fig. 1. 25OHD can enter the renal tubuli as free [not bound to the vitamin D–binding protein (DBP) or not bound to proteins in general] via the bloodstream or as a complex with DBP by uptake mediated by megalin/cubulin after filtration in the glomerulus. Most other cells only have access to free 25OHD except maybe for a few cells with low expression of megalin (placenta and parathyroid cells). Therefore, free 25OHD and the extrarenal expression of CYP2B1 define the local production of 1,25(OH)_2_D outside the kidney. Only kidney-produced 1,25(OH)_2_D can be exported to the bloodstream. Free 1,25(OH)_2_D then can gain access to the target tissues, activate the VDR, and thereby regulate gene transcription. A very large number of genes (∼3% of the human genome) are under the direct or indirect control of the active hormones, suggesting a broad spectrum of activities (Table 1). 1,25(OH)_2_D may also activate nongenomic pathways either by binding to the VDR or another receptor (1) located in lipid rafts in caveolae, thereby transiently regulating ion channel activity (especially chloride and calcium channels), kinases, and phosphatases (2). The biological consequences of VDR activation are well understood and described in greater detail in this review. The biological implications of nongenomic actions for tissue or whole-body physiology are poorly understood. We mention two examples. First, the *in vivo* administration of a bolus of 1,25(OH)_2_D can rapidly and transiently increase intestinal calcium absorption in chicks. Second, the absence of FAM57B2, a membrane receptor for 24R,25(OH)_2_D, results in a transient delay in fracture healing, similar to the same phenotype as found in *cyp24a1*-deficient mice (3). We will survey the well-documented and potential benefits of vitamin D and the risks associated with vitamin D deficiency with the goal of identifying a vitamin D status that is effective and safe in protecting health globally. First, we summarize data related to metabolic bone diseases and thereafter summarize the present state of the art regarding the possible extraskeletal actions of the vitamin D endocrine system. Throughout this review, preclinical data are briefly summarized, whereas human data are discussed in somewhat greater detail. Observational studies are briefly summarized whereas we focus more on Mendelian randomization (MR) trials and randomized controlled trials (RCTs) and their meta-analyses. Indeed, apart from an ongoing discussion about the relative value of observational vs RCTs (4, 5), most governmental authorities consider RCTs the most convincing way to demonstrate the role of vitamin D in health. There is increasing attention to the use of MR studies to evaluate the lifelong consequences of genetically predisposed lower or higher serum 25OHD concentrations and health outcomes (6).

**Table 1.** Comparison Between the Vitamin D and Thyroid Endocrine Systems

	Vitamin D Endocrine System	Thyroid Endocrine System	Comment
Substrate	Dietary vitamin D_3_/D_2_ or 7DHC and UV-B light	Iodide	• For both systems the availability of the substrate is irregular, as most food items contain little substrate (D_3_/D_2_ or iodide)
			• UV-B light for vitamin D synthesis is also dependent on geographic, seasonal, climatic, and cultural factors
			• Around the world the total supply of both substrates varies from very low to very high
Storage of inactive precursor	Vitamin D in fat, liver, muscle	Tg in thyroid colloid	• Storage of vitamin D in different tissues and its dynamics back to the plasma pool are poorly understood
			• Active accumulation of iodide in Tg allows for storage of iodide for several weeks or months of thyroid hormone synthesis
First metabolic activation into plasma prohormone	Conversion of vitamin D into 25OHD by CYP2R1 and other CYPs	Digestion of Tg into T_4_	• Synthesis of 25OHD is poorly regulated except by the dynamics of CYP2R1 and other CYPs with little or no feedback regulatory control
			• Conversion of Tg into T_4_ is regulated by endogenous feedback systems (TRH–TSH–TSH receptor)
			• Both mechanisms create a large plasma pool of prohormone with long half-life (2 wk for 25OHD and 1 wk for T_4_)
Second metabolic activation/inactivation	25OHD can be activated by CYP27B1 into 1,25(OH)_2_D or inactivated into 24R,25(OH)_2_D by CYP24A1	Tg or T_4_ activation into T_3_ or inactivation into reverse T_3_	• Activation or inactivation is strongly controlled by hormones
			• For vitamin D, PTH and FGF23 are the main regulators
			• TSH is the main regulator for thyroid hormone metabolism
Tissues involved in activation/inactivation	Kidney is the major regulator for synthesis of 1,25(OH)_2_D for export to plasma; many other tissues can produce this hormone for local autocrine/paracrine action	Thyroid gland and muscle are major tissues of synthesis of T_3_ for export to plasma pool; most tissues have local deiodinases for activation/inactivation of T_4_	
Control of metabolism	PTH and FGF23 are major regulators for metabolism in kidney; several cytokines regulate synthesis in other tissues	TSH is major regulator of metabolism in thyroid gland; other hormones and cytokines regulate iodinase activity in other tissues	
Hormone action			
Binding to NR as ligand of NR	1,25(OH)_2_D binding to VDR	T_3_ binding to TR	• Both hormones bind with high affinity to their respective receptors whereas the circulating hormone precursors have a much lower affinity for the receptors
			• VDR and TR are both members of the large family of nuclear receptors and use the same heterodimer partner (RXR) and coactivators or repressors, and they bind to similar hexanucleotide sequences in DNA (direct repeat hormone-responsive elements) separated by three or four nucleotides, respectively
Genomic action	Gene regulation	Gene regulation	• Both hormones regulate a very large number of genes (>1% of the human genome), suggesting a broad spectrum of activities, using a complex gene transcription mechanism similar to ligands of other NRs
Nongenomic action	Second signaling pathways	Second signaling pathways	• As for other hormones, nongenomic actions are operational but their clinical implications are incompletely understood

The origin of the substrate is irregular for most subjects. Therefore, evolution created a tissue storage of inactive precursors and a large circulating pool as a prohormone (25OHD and T_4_, respectively) with a long half-life. Thereafter, the prohormone can either be activated or inactived either for systemic transport or for local autocrine/paracrine actions. Both systems use a gene transcription regulatory mechanism based on strongly related nuclear receptors and use the same heterodimer partner (RCR) and similar hormone response elements to regulate a very large number of genes.

Abbreviations: 7DHC: 7-dehydrocholesterol; FGF23, fibroblast growth factor 23; NR, nuclear receptor; RXR, retinoic acid receptor; T_3_, triiodothyronine; T_4_, thyroxine; Tg, thyroglobulin; TR, thyroid hormone receptor.

**Figure 1.**
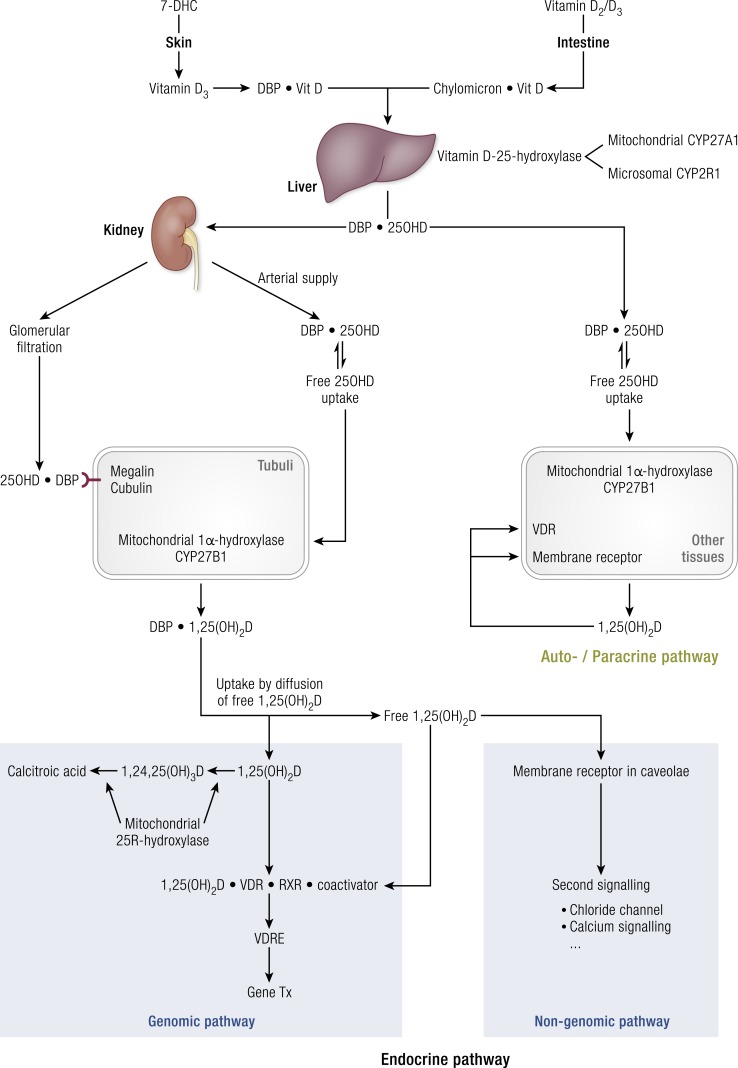
Metabolism and action of vitamin D and its metabolites, with special focus on renal and extrarenal production of 1,25(OH)_2_D and the genomic or nongenomic pathways of vitamin D action.

Indeed, serum 25OHD concentrations are under genetic control, and several large-scale genome-wide association studies (GWASs) have consistently identified a number of single nucleotide polymorphisms (SNPs) around genes involved in vitamin D synthesis, metabolism, and transport, which alone or in combination modify serum 25OHD concentrations (7, 8). Such data allow identifying subjects with genetically predisposed serum 25OHD significantly lower or higher than the mean of the population. Therefore, large-scale genetic studies can define whether such genetically predisposed lower serum 25OHD concentrations are linked to specific health outcomes, thereby avoiding the problem of reverse causation, residual confounding, or limited duration of observation or interventions. MR studies dealing with vitamin D status, however, are limited by the relatively low predicted differences in serum 25OHD (usually <5% of total variation). Additionally, such studies evaluate only linear correlations and cannot detect effects above or below a certain threshold (6).

Finally, we present the major outstanding research questions. For the purpose of this review, we define vitamin deficiency by serum 25OHD <50 nmol/L (or 20 ng/mL) and severe vitamin D deficiency by values <30 nmol/L (or 12 ng/mL). A more extensive analysis of the assay methodology to estimate the vitamin D status (9) and the definition of vitamin D status (10) is discussed in other recent studies (11).

## Vitamin D Is Essential for Skeletal Health

### Preclinical data

#### Growth plate

The abnormal structure of the growth plates is one of the major clinical, radiological, and histological hallmarks of rickets observed in humans and mice with vitamin D deficiency or inactivation of the VDR or 1*α*-hydroxylase (CYP27B1) (12–14). This phenotypic characteristic, however, does not result from direct VDR actions in growth plate chondrocytes but from the hypophosphatemia that decreases the cell death of hypertrophic chondrocytes (15, 16) and from hypocalcemia blocking chondrocyte differentiation. The growth plate structure, along with secondary hyperparathyroidism and hypophosphatemia, can be normalized by a very high oral calcium supply or by intravenous calcium administration, as demonstrated in humans and experimental animal models (17). Indeed, chondrocyte-specific deletion of *Vdr* or *Cyp27b1* in mice does not generate a rachitic growth plate (18, 19). Alternatively, local production of hormonal 1,25(OH)_2_D (or calcitriol) or *Vdr* action in chondrocytes has temporary paracrine and endocrine actions. Mouse genetic studies show that the absence of vitamin D signaling specifically in growth plate chondrocytes generates a transient increase in bone mass related to decreased production of pro-osteoclastogenic factors by mutant chondrocytes. Additionally, serum phosphate levels are transiently increased, as FGF23 production by osteocytes and osteoblasts is decreased (18, 19).

#### Bone and bone cells

In conditions with defective systemic vitamin D signaling, the effects of vitamin D on bone are largely indirect and caused by a negative calcium balance resulting from reduced vitamin D action in the intestinal mucosa. This conclusion is based on several observations. First, the osteomalacia of mice with systemic inactivation of *Vdr* signaling can be rescued by a diet containing high calcium and lactose, similar to the restoration of the growth plate phenotype (17, 20). More precisely, the rescue in *Vdr*-null mice was complete, but not in *Cyp27b1* mice (12, 21), and an explanation for this different response may be found in ligand-independent effects of the Vdr or differences in genetic or housing protocols. Second, mice with global *Vdr* inactivation and selective reintroduction of the Vdr in either the whole intestine (22) or only of the distal part of the intestine do not develop a skeletal phenotype (23). Finally, selective deletion of the *Vdr* in the intestine generates severe osteomalacia and decreased bone mass (24).

These data clearly indicate that the intestine is the primary target tissue for vitamin D action in calcium homeostasis and show that intestinal vitamin D signaling is necessary for adequate active transcellular and possible paracellular calcium transport (12, 20).

During a negative calcium balance, but with a normal active vitamin D system in mature osteoblasts and osteocytes, vitamin D signaling in bone cells has a role in maintaining serum calcium homeostasis by increasing bone resorption and impairing bone mineralization (24). These findings demonstrate that under these conditions, the vitamin D endocrine system primarily defends a normal serum calcium homeostasis, if needed, at the expense of bone.

During a normal calcium balance, implying normal intestinal calcium absorption, the absence of the *Vdr* in osteoblasts, osteocytes, or osteoclasts does not phenocopy rickets and osteomalacia, indicating a rather redundant role of the vitamin D endocrine system in bone cells (24–26). Nevertheless, mouse genetic studies have shown that both the absence and overexpression of the *Vdr* in osteoblasts increase bone mass modestly, in part by decreasing the expression of pro-osteoclastogenic factors (26, 27), but the exact mechanisms are incompletely understood. Moreover, the presence of the *Vdr* in osteoblasts mediates the bone anabolic action (in mice) of some vitamin D analogs (25). Finally, the roles of local production of 1,25(OH)_2_D in bone cells and its autocrine and paracrine effects have been suggested by *in vitro* data, but they need to be confirmed by *in vivo* experiments.

### Human data

#### Pathophysiology

**Figure 2.**
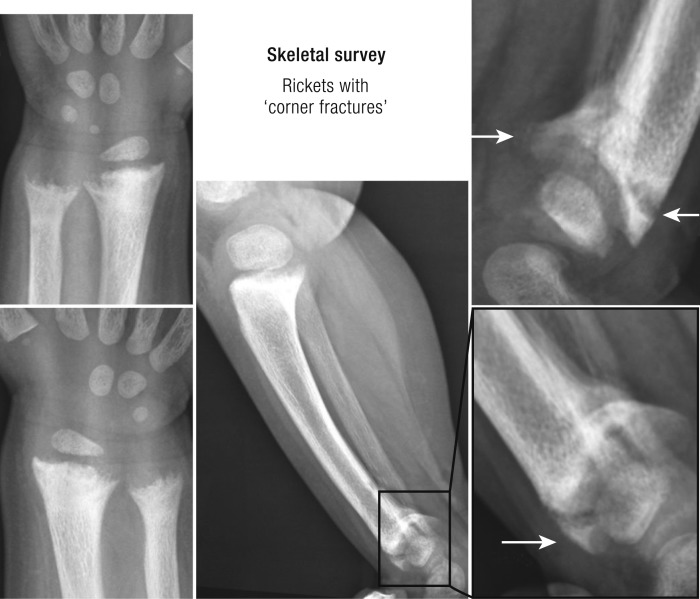
Radiologic image of nutritional rickets. A radiologic image of a 19-mo-old child with nutritional rickets is shown. The child was born from Indian parents, living in Australia, after a normal pregnancy of 40 wk and received exclusive breastfeeding for 18 mo without vitamin D supplementation. Height and weight were around the 50th percentile. Medical attention was asked because of genu varum and delayed walking. Serum calcium (2.01 mmol/L; 2.10 to 2.65) and phosphate were slightly decreased. Serum 25OHD was <18 nmol/L and alkaline phosphatase and PTH (126 pmol/L; 1.0 to 7.0) levels were high.

Severe vitamin D deficiency (30 nmol/L) in infants or children can cause rickets (28–30) (Fig. 2). The vitamin D endocrine system plays a limited role in prenatal life but becomes active shortly after birth. Because fetal serum 25OHD reflects that of the mother, maternal vitamin D deficiency increases the risk of nutritional rickets and hypocalcemia in the first months of life. The vitamin D content of mother milk or nonfortified cow milk is low, and exposure of infants or small children to direct sunlight may be dangerous. The incidence of rickets in Western populations is low owing to the widespread vitamin D supplementation of 400 IU/d in most Western countries for many decades. When this is not done, for example, in the case of a macrobiotic diet or in children of non-Western immigrants, the incidence of rickets increases sharply (31, 32). Low adherence rates to vitamin D supplementation in the very young in the United Kingdom have been leading to a resurgence of rickets and even casualties due to cardiomyopathy. Part of this is due to an increase of the nonwhite population, rising from 5.9% in 1991 to 14% in 2011 (33, 34). Systematic daily supply of 400 IU of vitamin D can prevent nutritional rickets, as was clearly demonstrated in Turkey where the incidence of rickets was reduced from 6% to 0.1% (35). However, rickets can still develop independent of vitamin D status with an intake of calcium of <250/300 mg daily (up to 12 months and after 12 months of age, respectively) (28, 36). Nutritional rickets is a risk factor for fractures in children and adolescents, and it can also have major consequences for tooth development (37). Despite well-established consensus guidelines on how to eliminate nutritional rickets, many infants and children are still at risk for this disease owing to the lack of implementation of simple interventions (28, 30).

The skeletal effects of moderate vitamin D deficiency in adults or elderly subjects are mainly caused by an increase of the serum PTH concentration, leading to high bone turnover and associated cortical bone loss (38). In the MORE study, the groups with vitamin D deficiency (serum 25OHD <25 nmol/L, n = 297 and 25OHD 25 to 50 nmol/L, n = 1721) both show significantly higher serum PTH (4.8 ± 2.2 and 4.1 ± 1.8 pmol/L, respectively) compared with people with 25OHD >50 nmol/L (n = 4982, serum PTH 3.5 pmol/L). Both groups show a significant decrease of serum PTH after treatment with vitamin D (17% and 12% lower serum PTH, *P* < 0.001), suggesting that serum PTH was elevated on an individual level in most patients (39). Very similar observations were made in the bazedoxifene trial in >7000 participants (40). This study shows thresholds for PTH and bone mineral density (BMD) at serum 25OHD of 50 or 75 nmol/L, respectively. The Longitudinal Aging Study Amsterdam (LASA) confirms these thresholds for PTH and BMD (41). Additionally, vitamin D deficiency may increase the risk of falls (as discussed below). Severe vitamin D deficiency may cause mineralization defects in some cases. An increase of osteoid volume (>5%) was observed in 10% of hip fracture patients having a serum 25OHD <30 nmol/L (42), and in a very large postmortem series, an osteoid volume >5% was observed in 4.8% of cases (43). This study caused serious discussion because of uncertainties about the accuracy of blood 25OHD measurement in postmortem samples and the use of histological criteria for osteomalacia, which do not correspond to the standard criteria (38, 44).

#### Epidemiologic studies

In the National Health and Nutrition Examination Survey (NHANES), an association between BMD and serum 25OHD was observed. BMD of the hip increased 0.06 g/cm^2^ in whites between 20 and 50 years of age when serum 25OHD increased from 20 to 90 nmol/L (45). In older persons, the BMD increase was somewhat less. In the LASA study, BMD of the hip increased 0.06 g/cm^2^ when serum 25OHD increased from 20 to 50 nmol/L (41, 45). A similar relationship was found in the bazedoxifene study (41). The LASA study also found an association between vitamin D deficiency and fractures. Serum 25OHD levels below or equal to 30 nmol/L were associated with an increased fracture risk [hazard ratio (HR), 3.1] in persons aged 65 to 75 years (4). Similarly, increased fracture risk was observed in subjects with the lowest vitamin D status in several long-term follow-up studies (46). Two recent RCTs confirmed a positive effect of vitamin D supplementation on BMD in subjects with a baseline serum 25OHD <30 nmol/L (47, 48).

#### MR studies

Two MR studies have evaluated the impact of genetically low serum 25OHD concentrations, as predicted by polymorphisms in four genes (DHCR7 and CYP2R1 involved in synthesis of vitamin D metabolites, and GC/DBP and CYP24R1 involved in transport and metabolism of vitamin D) (7, 10). A small-scale [Table 2 (49–86)] Chinese study of 1824 postmenopausal women did not find an effect of genetically predicted lower serum 25OHD on BMD at lumbar spine or femoral neck (75). A much larger study in subjects of European descent similarly did not find an effect of the same polymorphism in four vitamin D–related genes on either BMD or ultrasound characteristics of bone (76).

**Table 2.** MR Studies: Vitamin D Status and Clinical Endpoints

Disease	Population	Polymorphism	Results
**→ Cancer**			
Chandler *et al.* (49)	European women	5 SNPs	All cancers: HR, 1.01 (NS)
	N = 23,293		Breast: HR, 1.02 (NS)
	Subgroup with validation SNPs–25OHD		Colon: HR, 1.06 (NS)
			Lung: HR, 1.00 (NS)
			Cancer deaths: HR, 1.00 (NS)
			11 nmol/L difference highest/lowest NSP score
Dimitrakopoulo *et al.* (50)	Genetic networks	4 SNPs	OR for predicted, 25 nmol/L
	GAME-ON Consortium		Difference
	GECCO Consortium		Colorectal cancer: 0.92 (NS)
	PRACTICAL Consortium		Breast cancer: 1.05 (NS)
	MR-based platform		Prostate cancer: 0.89 (NS)
	N = 70,563 cancer cases		Lung cancer: 1.03 (NS)
	N = 84,418 controls		
Wang *et al.* (51)	Women of African diaspora	2 SNPs vitamin D status SNP for pigmentation (TYRP1)	Cancer: NS
	1657 Cases of breast cancer		
	2029 Controls		OR, 1.54 (*P* < 0.08)
	Total of 3686 participants		
Ong *et al.* (52)	N = 31,719 whites (10,065 cases vs 21,654 controls)	3 SNPs	OR per 20 nmol/L
	Ovarian cancer (all types)		Lower 25OHD:
	High-grade ovarian cancer		OR, 1.27 (1.06–1.51)
			OR, 1.54 (1.19–2.01)
Trummer *et al.* (53)	Prostate cancer	1 SNP	NS
Theodoratou *et al.* (54)	Colorectal cancer	1 SNP	NS
	2001 Cases (Scotland)		
	Comments: higher measured serum 25OHD associated with lower colorectal cancer incidence		
Dudding *et al.* (55)	Oral or oropharyngeal cancer	5 SNPs	OR, 1.01 (NS) (confirmed in validation cohort of 585 cases from UK Biobank)
	N = 5133		
	N = 5984 controls (Europe, North and South America, all with >70% European origin)		
Sun *et al.* (56)	Lung cancer	3 SNPs	Genes explained 3.4% of 25OHD HR, 0.96 (NS)
	N = 676 cases		
	N = 54,580 controls		
**→ Neurologic diseases**			
*Parkinson disease*			
Larsson *et al.* (57)	5333 Parkinson cases	4 SNPs	OR for 10% lower predicted
	12,019 Controls		Serum 25OHD, 0.98 (NS)
*Alzheimer’s disease*			
Mokry *et al.* (58)	Internal genomics of AD Consortium	4 SNPs from	1 SD lower predicted
	N = 17,008 cases	SUNLIGHT Consortium	25OHD: OR for AD,
	Versus 37,154 controls	(N = 33,996)	1.25 (*P* < 0.02)
	CaMos; n = 2347 cases		
Taylor *et al.* (59)	Schizophrenia		NS
*MS*			
Gianfrancesco *et al.* (60)	Early onset	3 SNPs	Higher predicted 25OHD:
	(Pediatric) MS		OR for MS, 0.72 (0.55–0.94)
	N = 394 United States vs 10,875 controls		
	N = 175 Sweden vs 5376 controls		
Mokry *et al.* (61)	Canada	4 SNP	1 SD predicted lower
	N = 33,996 and 2347 cases?		Log 25OHD: OR for MS, 2 (1.7–2.5) (*P* = 10^−12^)
Rhead *et al.* (62)	N = 1056 cases vs 9015 United States controls (non-Hispanic whites)	3 SNPs	OR for highest predicted
	N = 6335 cases vs 5762 Sw controls		25OHD, 0.85 (0.76–0.94) (*P* = 0.003)
**→ Diabetes and metabolic syndrome**			
Cooper *et al.* (63)	Type 1 diabetes	5 SNPs (including CYP27B1)	OR, 1.07 cases/controls (*P* = 0.007)
	n = 720 + 8517 cases		OR, 1.10 family study (*P* = 0.001)
	n = 13,438 controls		
	(White Europeans)		
Afzal *et al.* (64)	96,423 Danish subjects with/without T2DM	SNPs in DHCR7 and CYP2R1	20 nmol/L genetically lower 25OHD
			OR for T2DM, 1.51 (DHCR7) (*P* = 0.04)
			OR for T2DM, 1.02 (CYP2R1) (NS)
Ye *et al.* (65)	T2DM	4 SNPs	NS for T2DM or fasting blood glucose
	28,144 cases T2DM		
	76,344 controls		
	Comments: lower measured serum 25OHD associated with risk of T2DM		
Vimaleswaran *et al.* (66)	Obesity	4 SNPs (vitamin D)	NS link with BMI significantly lower measured 25OHD concentrations when SNPs for higher BMI
	21 Cohorts of European origin	12 SNPs (BMI)	
	N = 42,024		
	Comment: genes for higher BMI are associated with lower measured serum 25OHD, but genes for lower 25OHD concentrations are not significantly associated with higher BMI		
Husemoen *et al.* (67)	Adiponectin as marker of metabolic syndrome	1 SNP (DBP/GC)	Genetically twofold higher 25OHD associated with 1.6-fold higher serum adiponectin
	6405 + 2656 Danish subjects		
	Comment: a 1.6-fold higher serum adiponectin level could explain a twofold lower risk for T2DM		
Wang *et al.* (68)	Nonalcoholic fatty liver disease	4 SNPs	NS
	9182 Subjects from East China		
	Comments:		
	• No link between eight SNPs predisposing for nonalcoholic fatty liver disease and measured serum 25OHD		
	• Link between four SNPs for vitamin D and serum 25OHD concentrations confirmed in Chinese subjects		
**→ Cardiovascular events**			
Manousaki *et al.* (69)	SUNLIGHT Consortium	4 SNPs	NS
	N = 33,996		
	Canadian artery disease		NS
	N = 22,233 cases vs 64,762 controls		
Brøndom-Jacobsen *et al.* (70)	Denmark	4 SNPs in two genes	NS
	N = 92,416 total participants		
	N = 14,455 with ischemic heart disease		Comparison: lower measured serum 25OHD Lowest/highest quartile: HR, 1.82 for ischemic heart disease (1.42–2.32)
	N = 7061 with myocardial infarction		
Leong *et al.* (71)	Cardiovascular and metabolic disease 2254 Canadian subjects Comments: no link between this SNP and fasting blood glucose, insulin, BMI, cardiovascular diseases, and stroke	1 SNP (DBP/GC)	NS
Ooi *et al.* (72)	Nonfasting remnant cholesterol concentration	4 SNPs	NS*
	85,869 Whites (Denmark) for lipoproteins		Measured nonfasting cholesterol remnants inversely associated with measured serum 25OHD
	25,862 Whites (Denmark) for 25OHD		Genes for higher cholesterol remnants associated with lower serum 25OHD
	Comments: genes related to higher nonfasting cholesterol remnants are associated with lower serum 25OHD concentrations and thus may partially explain epidemiologic links between lower vitamin D status and cardiovascular risks and diseases and higher prevalence of low-grade inflammation		SNPs in vitamin D–related genes are related to serum 25OHD but only marginally with measured serum HDL
Vimaleswaran *et al.* (73)	Hypertension	2 SNPs (DHCR7 and CYP2R1)	10% Genetically higher serum 25OHD is associated with 0.3 mm Hg lower diastolic and systolic blood pressure and lower risk for hypertension
	142,255 Danish subjects		
Skaaby *et al.* (74)	Cardiovascular risk factor (lipid profile)	SNPs for fillagrin	Loss of fillagrin mutations result in 10% higher measured serum 25OHD (possibly related to higher UV-B–induced efficacy in vitamin D production) and better lipid profile (higher high-density lipoprotein, lower low-density lipoprotein, and lower very-low-density lipoprotein and triglycerides)
	11,983 Subjects of North European origin		
**→ Bone: BMD**			
Li *et al.* (75)	1824 Chinese postmenopausal women	4 SNPs	NS
	Serum 25OHD (measured) positively associated with BMD of lumbar spine (*P* = 0.003), femoral neck (*P* = 0.006), and total hip (*P* = 0.005)		
Larsson *et al.* (76)	2 Cohorts of European descent	5 SNPs linked to four vitamin D–related genes	NS
**→ Eye: myopic refractory disease**			
Cuellar-Partida *et al.* (77)	CREAM = 33,382 European and 8376 Asian participants	4 SNPs	Refractory error: 0.01 to −0.02 diopters per 10 nmol/L predicted increase in serum 25OHD (NS)
**→Immunological events**			
*Asthma and atopic dermatitis*			
Manousaki *et al.* (78)	SUNLIGHT, GABRIEL, and EAGLE (eczema) Consortia	4 SNPs	OR for disease per SD
	Asthma (N = 146,761)		OR, 1.03 (NS)
	Childhood onset asthma (N = 15,008)		OR, 0.95 (NS)
	Atopic dermatitis (N = 40,835)		OR, 1.12 (NS)
	Elevated IgE level (N = 12,835)		Effect size, 0.40 (NS)
Mao *et al.* (79)	Asthma GABRIEL database of 10,363 European cases vs 16,110 controls	4 SNPs	NS
*Inflammation (C-reactive protein)*			
Liefaard *et al.* (80)	Rotterdam study on 9649 participants Measurement of C-reactive protein as marker of inflammation	4 SNPs	NS
*RA*			
Viatte *et al.* (81)	RA outcome 493 + 2924 cases of RA (United Kingdom)	4 SNPs	NS
	Comments: study of outcome (signs and symptoms) of RA and not of prevalence or incidence of RA		
**→ Skin aging**			
Noordam *et al.* (82)	Rotterdam and Leiden studies	? SNPs	NS
	N = 3831 and 661		
	Facial skin aging features		
	Perceived age, wrinkling, pigmented spots		
	Serum measured 25OHD associated with skin aging: higher serum 25OHD, higher skin aging (*P* > 10^−6^)		
**→ Mortality**			
Ordóñez-Mena *et al.* (83)	German older adults (ESTHER)	4 SNPs	NS
	N = 8417		
	2 SNPs associated with lower serum 25OHD		
	Lower serum 25OHD associated with higher mortality		
Afzal *et al.* (84)	3 Danish cohorts	4 SNPs in two genes	
	N = 95,766		
	Follow-up 9–19 y		
	Genetically low serum 25OHD: per 20 nmol/L lower serum 25OHD		
	All-cause mortality:		1.3 (1.05–1.61)
	Cardiovascular mortality:		NS
	Cancer mortality:		0.43 (1.02–1.99)
	Additionally measured serum 25OHD in 35,334 subjects		
	Per 20 nmol/L lower measured serum 25OHD		
	All-cause mortality:		1.19 (1.14–1.25)
	Cardiovascular mortality:		1.18 (1.09-1.28)
	Cancer mortality:		1.12 (1.03-1.22)
Trummer *et al.* (85)	Mortality in 3316 German adults undergoing a coronary angiography (age 63 y) and followed up for 10 y	4 SNPs	NS
**→ Kidney function**			
Teumer *et al.* (86)	Glomeral filtration rate 16,442 + 5123 objects of European ancestry	3 SNPs	Negative effects of higher 25OHD on estimated glomerular filtration rate (*P* = 0.003)
**→ Conclusions**	1. Genetically low 25OHD associated with all-cause and cancer mortality		
	2. Observational low 25OHD associated with all-cause, cardiovascular, and cancer mortality		

Overview of MR studies dealing with polymorphism in genes related to vitamin D synthesis, transport, or metabolism and serum 25OHD vs different biological endpoints or diseases. Unless mentioned otherwise, SNPs mentioned in this table refer to polymorphism in genes for 7-dehydrocholesterol-reductase (DHCR7), CYP2R1 or 25-hydroxylase, DBP/GC, the major serum transport protein for all vitamin D metabolites, and CYP24A1, the major catabolizing enzyme for 25OHD and 1,25(OH)_2_D.

Abbreviations: NS, not significant; T2DM, type 2 diabetes mellitus.

#### RCTs

Many RCTs have been performed with vitamin D, usually combined with calcium, on BMD and fractures as outcome criteria (87). The effects on BMD are best visible at the femoral neck, +0.8% on average (range, 0.2% to 1.4%) according to a recent meta-analysis (88). However, this meta-analysis did not include the Lyon clinical trial (89). In this trial in a very vitamin D–deficient population, the difference in total hip BMD between vitamin D and the control group was 7.3%.

In the large VIDA study of adults in New Zealand (mean age, 69 years; mean baseline serum 25OHD, 56 nmol/L) (48), a modest increase in BMD at the femoral neck (+0.5%) was observed overall in the group treated with 100,000 IU of vitamin D_3_ per month for 2 years. In the subgroup with a baseline serum 25OHD <30 nmol/L, BMD remained stable during 2 years in vitamin D–supplemented subjects, whereas a 2% decrease was observed in the control group. The effect of vitamin D on fracture incidence was studied in at least 19 RCTs. In these trials, vitamin D was given with different intervals, from daily to once per year. In five trials, vitamin D was given alone. In two of these, a significant decrease of fracture incidence was observed, either with annual injection or with a 4-monthly oral dose (90, 91). The three other trials with vitamin D alone were negative (92–94). In 13 RCTs, vitamin D and calcium were combined. The greatest effect was observed in the first trial in a very deficient French nursing home population (mean age, 84 years) treated with vitamin D_3_ (800 IU/d) and calcium (1200 mg/d) vs double placebo. In this trial, a considerable and significant decrease of hip fracture incidence (−20%) and other fracture incidence (−25%) was observed (89), as well as an increase of BMD at the hip of 6% (see above). In two other trials the combination of vitamin D and calcium showed a significant decrease of fracture incidence (95, 96). In two other trials the combined therapy showed a borderline effect, the first in a similar French nursing home population (*P* = 0.07 for nonvertebral fractures) (97), and the second from the Women’s Health Initiative (98) (hip fractures, intention-to-treat: HR, 0.88; 95% CI, 0.72 to 1.08; per protocol analysis in adherent subjects: HR, 0.71; 95% CI, 0.52 to 0.97]. Because of the coadministration of vitamin D and calcium, it is not possible to define the relative contribution of vitamin D and calcium supplementation. Six trials did not show a significant effect of vitamin D and calcium on fracture incidence (91, 99–101). Two RCTs, however, showed an increase in fracture incidence, both employing a very high single dose (300,000 and 500,000 IU) once per year (102, 103). One of these also showed an increased risk of falls (102). In the most recent VIDA study (104), no effect of vitamin D supplementation on fractures or falls risk was observed during a 3.4-year follow-up of New Zealand adults (mean age, 69 years) with a baseline serum 25OHD of 63 nmol/L. Whether this null result was due to the good vitamin D status at baseline, the use of monotherapy with vitamin D without calcium supplementation, or the high intermittent dose of 100,000 IU/mo is unclear. Intermittent high-dose vitamin D may paradoxically and transiently increase the fracture risk (and falls; see below) (102, 103).

#### Meta-analyses

Many meta-analyses have evaluated the effect of vitamin D on BMD and fracture incidence (88, 105–109). The outcomes of meta-analyses vary greatly (108). In general, the meta-analyses show that the effect of vitamin D is greater when (1) given to older (70 to 80 yrs or ≥80 years) than to younger subjects (60 to 70 years), (2) given to those in a residential care setting than to independently living elderly (109), or (3) the daily dose is at least 800 IU or when baseline serum 25OHD is low. The Cochrane systematic review and meta-analysis stated that vitamin D alone is unlikely to be effective in preventing hip fracture or any new fracture. However, it also showed that combined vitamin D and calcium supplementation induces a 16% decrease in hip fracture risk, a 14% decrease in new nonvertebral fracture risk, and a 5% decrease in risk for any fracture (105). To avoid one hip fracture, 1000 older persons must be treated for 1 year. Treatment is much more efficacious in high-risk persons, such as the institutionalized, where ∼110 persons must be treated for 1 year to save one hip fracture. This number decreases further when other nonvertebral fractures are included. Side effects of calcium and vitamin D include hypercalcemia (rare) and renal stones. Mortality decreases by 6%, but this was not significant (105). One meta-analysis concluded that the effect was trivial as the decrease in fracture incidence was not >15% (110). However, it may be argued that a 10% to 15% decrease of fracture incidence is considerable, as this therapy can be implemented in a very large number of subjects at risk, at very low cost and with limited side effects.

#### Research agenda

Despite major progress during the last decades in understanding the role of vitamin D and its metabolites on calcium and bone homeostasis, many important questions remain incompletely answered. There is at least one missing player in our understanding of the role and mechanism of action of the vitamin D system on transepithelial calcium transport in the intestine. The precise role of the vitamin D endocrine system in other calcium-transporting or calcium-sensing systems, such as kidney, placenta, breast, and parathyroid glands, is also still incomplete. The role of 1,25(OH)_2_D production in calcium-transporting or calcium-sensing tissues and its autocrine/paracrine effects should also be defined. Additionally, the precise contribution of the vitamin D system on overall phosphate homeostasis is incompletely understood. A better understanding of the risk factors for the development of nutritional rickets beyond vitamin D deficiency is required, for example, dietary calcium intake, iron deficiency, and genotype. The effect of vitamin D status on skeletal development during fetal life, childhood, and adolescence requires greater clarification. Most RCTs used daily doses of vitamin D between 400 and 1200 IU. The effect of higher doses is not well known. The effect of vitamin D metabolites or analogs on bone structure, turnover, and fracture incidence is not clear. Most trials did not select participants with a low serum 25OHD level. Forthcoming trials should select participants based on low baseline serum 25OHD. Individual participant data meta-analyses should be performed selecting vitamin D–deficient subjects only. It may be worthwhile to model the effects of vitamin D supplementation according to age, sex, residence, baseline serum 25OHD, vitamin D dose, and the addition of calcium supplements. Additionally, specific risk groups should be defined for vitamin D supplementation to prevent fractures. To improve the efficacy of vitamin D, the treatment should be targeted to the most vulnerable groups, with the institutionalized group ranking highest (89, 105, 108).

### Conclusions

Vitamin D deficiency increases serum PTH, but most vitamin D–deficient subjects do not have PTH concentrations above the normal range. This results in progressive bone loss and, when severe, also mineralization defects. Epidemiologic studies show that vitamin D deficiency is associated with lower BMD and fractures. These consequences can be avoided by modest doses of vitamin D and calcium supplements (28, 35). RCTs have shown that vitamin D decreases the incidence of hip fractures and other nonvertebral fractures by ∼15%, with the effect being greater in the 80+ years of age and 70 to 80 years of age persons than in persons aged 60 to 70 years, in the institutionalized group than in community living elderly, when combined with calcium and when compliance is >80%. Vitamin D supplementation should be advised in all institutionalized and frail older persons. There is great unanimity that serum 25OHD concentrations <30 nmol/L should be corrected. Serum 25OHD levels <50 nmol/L should be avoided. For subjects with limited exposure to sunlight this requires a daily vitamin D intake of ∼800 IU/d. This advice is generally in line with most governmental guidelines (111), except for the UK Scientific Advisory Committee on Nutrition (29) who recommended serum 25OHD concentrations >30 nmol/L and a vitamin D intake of 400 IU/d for all subjects of whatever age at risk for vitamin D deficiency. This conclusion also does not contradict the conclusion of the United States Preventive Services Task Force dealing with a younger community dwelling population (mostly postmenopausal women) with a much better vitamin D status than the elderly or institutionalized subjects (112).

## Extraskeletal Actions of Vitamin D

The potential extraskeletal actions of vitamin D have generated considerable excitement during the last couple of decades, with a rapidly expanding number of studies using cell-based experiments and preclinical models of diseases. These studies were spurred in part by observations that both the VDR and CYP27B1 are present in a large number of cells and tissues not related to the classical target tissues for vitamin D (113). Additionally, many observations from gene expression profiling studies indicate that 1,25(OH)_2_D regulates the expression of numerous genes (from zebrafish to mice and humans) unrelated to calcium homeostasis (114). It is fair to say that the position of the Institute of Medicine committee, as well as nearly all official guidelines (111), of not considering a potential role for vitamin D extraskeletal health (115) was greeted with some consternation among enthusiasts for its “nonclassical” actions. Differences in opinion have led to extensive and lifely debates in journals and handbooks (10, 116). Therefore, we critically assess the accumulating preclinical and clinical evidence for a role of vitamin D signaling in physiological systems independent of calcium homeostasis.

### Skin as origin and target of vitamin D

#### Preclinical data

**Figure 3.**
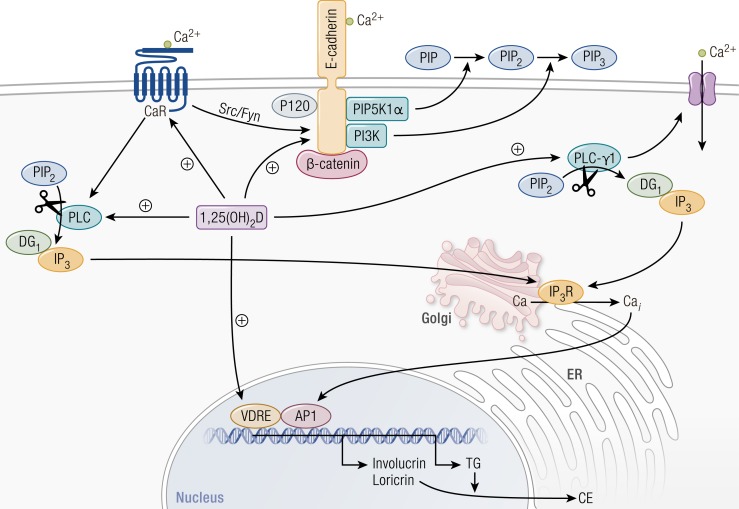
Regulation of keratinocyte differentiation by calcium and 1,25(OH)_2_D. Calcium and 1,25(OH)_2_D interact to regulate keratinocyte differentiation at multiple steps. 1,25(OH)_2_D acts via its nuclear hormone receptor, VDR, to directly regulate gene transcription. Among the genes that it regulates are involucrin and loricrin, which encode major constituents of the cornified envelope (CE) as well as transglutaminase (TG) that crosslinks these proteins and others to form the CE. Although the effects of calcium on gene transcription do not appear to be direct, calcium is likely to act at least in part through protein kinase C, which phosphorylates and so activates transcription factors of the AP-1 family critical for the induction of these genes. Not shown is that 1,25(OH)_2_D also induces genes that encode enzymes that produce the long-chain lipids required for “waterproofing” the CE. 1,25(OH)_2_D also induces the calcium sensing receptor (CaR) that responds to extracellular calcium by activating phospholipase C (PLC). PLC, by cleaving phosphatidylinositol bisphosphate (PIP_2_), releases two important signaling molecules: diacylglycerol (DG) and inositol trisphosphate (IP_3_). The latter releases calcium from intracellular stores such as the endoplasmic reticulum (ER) and Golgi through the IP_3_ receptor (IP_3_R). DG works in conjunction with calcium to activate protein kinase C. 1,25(OH)_2_D induces both the *β* and *γ* forms of PLC, but calcium is required for their activation. The CaR also activates Src/Fyn, which phosphorylate the catenins, including *β*-catenin, to enable their binding to and formation of the E-cadherin complex in the membrane. Both calcium and 1,25(OH)_2_D are essential for the formation of this complex: 1,25(OH)_2_D induces E-cadherin, whereas calcium promotes its translocation to the membrane. Not shown is that *α*-catenin binds to *β*-catenin, linking the E-cadherin/catenin complex to the cytoskeleton, critical for cell migration. The E-cadherin/catenin complex also contains two enzymes, phosphatidylinositol phosphate 5 kinase 1*α* (PIP5K1*α*) and phosphatidylinositol 3 kinase (PI3K), that sequentially phosphorylate PIP to PIP_2_ to PIP_3_. PIP_3_ is the major activator of PLC-*γ*1 during keratinocyte differentiation, which in addition to promoting the cleavage of PIP_2_ to IP_3_ and DG also activates at least one of the calcium channels, TRP3. [Reproduced with permission from Bikle DD. Vitamin D, Calcium. and the Epidermis. In: Feldman D, Wesley Pike J, Bouillon R, *et al.*, eds. Vitamin D. 4th ed. London: Academic Press; 2018:527-544.]

Vitamin D is produced mainly in the epidermis, where 7-dehydrocholesterol is converted to previtamin D_3_ under influence of UV-B and subsequently isomerized to vitamin D_3_. Vitamin D plays important intracrine, autocrine, and paracrine actions in the epidermis (Fig. 3) (117). Indeed, the dominant cells of the skin, the keratinocytes, are able to produce the active hormone 1,25(OH)_2_D, by their own 25-hydroxylase (CYP27A1) (118) and 1*α*-hydroxylase (CYP27B1) enzymes (119). Moreover, keratinocytes express the VDR, which is most abundant in the stratum basale and in the stem cells of the hair follicle (120). The active vitamin D locally produced does not appear to contribute to the circulating levels under normal circumstances, but it is involved in epidermal differentiation and proliferation, wound response, and tumorigenesis, acting on keratinocytes and their neighboring cells.

The *Vdr*-null mouse shows signs of disrupted epidermal differentiation (namely, low levels of loricrin, involucrin, and profilaggrin), which can be partially rescued with a high calcium diet. In *Cyp27b1-*null mice, the disorders in epidermal differentiation were not reversed with a high calcium diet (121). *In vitro* and *in vivo* studies revealed that 1,25(OH)_2_D and calcium stimulate keratinocyte differentiation in a synergic and somehow redundant way at different levels by: (1) transcriptional control of cell cycle regulatory proteins; (2) stimulation of proteins crucial for cornified waterproof envelope formation; (3) induction of the E-cadherin/*β-*catenin complex, required for epidermal differentiation and keratinocyte cell-to-cell adhesion (122); and (4) maintenance of calcium gradient along epidermis layers, which is critical for the correct differentiation process from the basal cells to the cornified cells (90–92, 122–124). VDR functions seem to be different in each layer of epidermis owing to the recruitment of specific modulators and coactivators (123).

As discussed below, innate and acquired immune responses are modulated by ligand-dependent VDR functions, and 1,25(OH)_2_D is involved in the process of wound repair and host protection. This is true also in the skin. Indeed, recent studies showed that in both *Vdr-*null mice and in vitamin D–deficient mice normal macrophage recruitment and formation of granulation tissue after a cutaneous injury are impaired. This phenotype is due to the disruption of VDR–TGF-*β* interaction, which seems to be crucial for wound response (124). VDR expression within the keratinocytes is also necessary for the re-epithelialization of wounds (125). Moreover, as discussed below, active vitamin D and its analogs have been shown to stimulate keratinocyte production of cathelicidin, an antimicrobial protein that improves angiogenesis and re-epithelialization in the skin after injuries as well as protection from invading organisms (126–128).

One of the most dramatic features of *Vdr-*null mice is the development of alopecia 4 to 10 weeks after birth, which cannot be rescued with a high-calcium diet. This particular type of alopecia is not observed in vitamin D–deficient mice or in *Cyp27b1-*null mice, suggesting that the action of the VDR in the hair follicle cycle is independent from its ligand (129). The VDR plays its role in the keratinocyte stem cell population located in the bulge of hair follicles, where it is crucial for the capacity to start a new hair cycle, after the first hair coat is lost. 1,25(OH)_2_D seems to be not involved, but cofactors and interacting molecules that can change VDR conformation and gene transcription need to be further investigated (130, 131). The VDR is also considered as a tumor suppressor in the skin. Indeed, in the absence of the VDR there is an increased susceptibility to chemical- or UV-induced tumors in animal models (132, 133). VDR activation controls the Wnt/*β-*catenin and sonic hedgehog pathways, which are overexpressed in VDR-null animals, leading to uncontrolled keratinocyte proliferation and tumor formation (133, 134).

#### Clinical data

Despite the abundance of preclinical data regarding vitamin D and skin cell interaction, it remains speculative whether low vitamin D status has a causative role in the pathogenesis of cutaneous diseases in humans or, as suggested for other diseases, it is a marker of ill health and inflammatory status (135).

##### Psoriasis.

The relationship between vitamin D and psoriasis has been extensively investigated. Indeed, it is well known that psoriasis is characterized by increased proliferation and decreased final differentiation of keratinocytes, but inflammation and autoimmune reactions also contribute to its clinical expression. No association was found between vitamin D intake (dietary and/or supplemental) and psoriasis incidence in a long-term study of >70,000 US female nurses enrolled in the Nurses’ Health Study (136). Early intervention studies with oral calcitriol therapy were found to improve the clinical course of psoriasis (137, 138). These benefits have been confirmed by more recent studies in a small cohort of psoriatic patients (139, 140), as well as by a study in which 0.25 μg of calcitriol daily was combined with acitretin (141). More recently, beneficial effects have been demonstrated with the topical application to psoriasis plaques of 1,25(OH)_2_D or analogs such as calcipotriol, maxacalcitol, tacalcitol, and hexafluoro-1,25(OH)_2_D. The calcitriol analog calcipotriol is ideal for topical treatment owing to its low affinity for DBP. Its efficacy and safety were demonstrated in prospective clinical trials (142). A recent meta-analysis confirmed that topical treatment with vitamin D analogs is as efficacious as topical corticosteroid therapy alone, with the advantage of the “steroid-sparing” effects (avoiding side effects of topical glucocorticoids), and that the combined therapy further increases the beneficial effects (143). Nowadays, the use of topical vitamin D derivatives is becoming the most common treatment of psoriatic lesions (144, 145). In combination with topical steroids, it may be particularly helpful in hard-to-treat areas of the skin (146).

##### Skin cancer.

The role of vitamin D in skin cancer is still a matter of controversy. There is no doubt that UV-B light, responsible for the local production of vitamin D, is carcinogenic. Vitamin D metabolites, however, also display anticarcinogenic activities by activating the repair mechanisms of DNA damage (147, 148). A photoprotective effect of 1,25(OH)_2_D and several even nongenomic agonists was also found in mice exposed to UV-B light (149). Several studies have investigated the association between vitamin D status and nonmelanoma skin cancer (NMSC). In a sample of the Osteoporotic Fracture in Men study, an inverse association between serum 25OHD levels and the incidence of NMSC was found (47% lower odds for men in the highest quintile compared with those in the lowest quintile) (150). However, another study in a health maintenance organization cohort showed that values of serum 25OHD >37.5 nmol/L were positively associated with an increased risk, even after adjustment for additional risk factors (151, 152). In these studies, the contribution of confounding factors such as UV-B exposure is difficult to analyze, and conclusive data are still lacking. As far as the relationship between vitamin D and melanoma is concerned, there is evidence of a protective effect of vitamin D_3_, but UV radiation, which is a principal source of vitamin D_3_, is mutagenic (142). In a *post hoc* analysis of a large sample (n = 36,282) of postmenopausal women, enrolled into the Women’s Health Initiative, there was no difference in the incidence of melanoma between women receiving daily low-dose vitamin D (400 IU) plus calcium (1000 mg) supplementation compared with placebo during a follow-up period of 7 years. Alternatively, the incidence of melanoma was lower in a group at high risk, with a history of NMSC, receiving calcium and vitamin D supplementation (153).

##### Miscellanea.

Despite the experimental evidence of an established role of vitamin D in hair follicle cycling, few clinical data are available in humans. A cross-sectional study on 296 healthy men did not show any association between severity and extent of baldness and serum 25OHD levels (154). Moreover, calcipotriol failed to improve alopecia in a placebo-controlled study on patients with scalp psoriasis (155). Finally, some data suggest a possible protective role of vitamin D in acne vulgaris but an adverse effect in patients with rosacea (156).

#### Genetic data

##### Psoriasis.

Two recent meta-analyses of the most widely studied *VDR* gene polymorphisms and psoriasis risk have been performed, which to some extent provide contradictory results. One study showed an association between Apa1 and Taq1 polymorphisms and psoriasis in whites (157), and the other concluded that no robust and reproducible association exists between Apa1, Bsm1, Fok1, and Taq1 and psoriasis, or, at most, only a weak association present only in specific ethnic groups (158). Previous studies found no association between VDR polymorphisms and the clinical response to topical vitamin D at least in Korean patients (159). Moreover, Kontula and Mee showed no association between *Bsm*I and response to calcipotriol therapy in psoriatic patients (160, 161). Alternatively, more recently, VDR Fok1 and Cdx2 polymorphisms were shown to influence the individual response to calcipotriol in monotherapy and when associated with steroid therapy in a Chinese population (162).

##### Skin cancer.

A study in a German population has shown an association between the Apa1 and Taq1 genotypes with basal cell carcinoma, but not with squamous cells carcinoma (163). Alternatively, the BSM1 genotype has been associated with both types of tumors. Several studies have looked at the association between VDR polymorphisms and melanoma. A relationship between sun exposure and VDR genotypes was evaluated in a case-control study in melanoma survivors. The authors found that the Bsm1 variant was associated with the occurrence of multiple primary melanoma (164). A meta-analysis of six studies that investigated the association between five VDR polymorphisms (*Taq*I, *Fok*I, *Bsm*I, *Eco*RV, and Cdx2) and the risk of melanoma also showed that the Bsm1 genotype was associated with an increased risk of melanoma development (165). Such an association has been recently confirmed by a meta-analysis, which reviewed 11 studies in European populations and analyzed the association between VDR *Fok*I, *Bsm*I, *Taq*I, *Apa*I, and *Eco*RV polymorphisms and susceptibility to melanoma (166).

So far, there is only one MR trial looking at vitamin D status and skin phenotype (Table 2). Noordam *et al.* (82) studied facial skin aging features in ∼4500 Dutch adults and found that higher measured serum 25OHD concentrations were associated with perceived age, skin wrinkling, and pigmented spots, but they genetically predicted that serum 25OHD was not linked with these skin characteristics. This seems to indicate that exposure to UV-B light rather than serum 25OHD concentrations are causally linked with skin aging.

### Conclusions

Skin provides an excellent and well-established example of the nonskeletal actions of vitamin D signaling. The skin is indeed the only tissue capable of synthesizing all important vitamin D metabolites and is also a major target for vitamin D and its metabolites. Skin keratinocytes express all enzymes of the vitamin D metabolic pathway and can produce hormonal 1,25(OH)_2_D_3_ in the presence of sufficient UV-B irradiation. 1,25(OH)_2_D_3_ thus produced controls keratinocyte proliferation and differentiation, as well as epidermal barrier integrity (167, 168). Clinically, topical application of vitamin D analogs shows clear efficacy in alleviation of symptoms of psoriasis, which likely arises from their effects on epidermal cell proliferation and, as discussed below, their anti-inflammatory properties.

UV-B light is essential for endogenous production of vitamin D, but the same wavelengths are also oncogenic. UV-B exposure indeed causes DNA damage and p53 expression, which are associated with systemic production of endorphins, which then can produce an addictive behavior [well documented in mice (169), but likely also in humans]. Moreover, mice deficient in vitamin D signaling overexpress the oncogenic transcription factor cMYC (170) and are more susceptible to skin carcinogenesis than are their wild-type counterparts (123). There is thus a very difficult trade-off between safe exposure to sunlight as to produce sufficient vitamin D while avoiding long-term risks of skin damage and skin cancer. VDR and 1,25(OH)_2_D action in the skin may generate some protective mechanisms against UV-B damage.

## Muscles and Falls

### Preclinical data

The expression of the VDR in muscle is hotly debated, as some experts could not detect VDR protein in adult multinucleated human or rodent skeletal muscle (171), whereas others found it to be widely expressed at the mRNA and protein levels (172, 173). In immature muscle cells or its stem cells, the VDR is probably expressed at a low level in comparison with the intestine (1000-fold lower mRNA and protein level), and it is probably absent or nearly so in mature multinucleated cells (174, 175). 1,25(OH)_2_D has clear antiproliferative effects on cultured muscle cells and regulates several genes involved in muscle cell maturation, including a negative regulation of myostatin (172, 176). Systemic *Vdr* knockout mice have smaller and immature skeletal muscle cells, especially of fast-twitch (glycolytic) type II fibers (172, 173, 176), and selective *Vdr* deletion in cardiomyocytes generates a clear phenotype of hypertrophy and fibrosis (see “Cardiovascular System” below).

### Human data

Observational data suggest that severe longstanding vitamin D deficiency is associated with muscle weakness and cardiomyopathy in infants (17). Such severe muscle weakness is also seen in patients with congenital absence of CYP27B1 or in patients with severe renal osteodystrophy. Rapid improvement of muscle function has been reported after vitamin D or 1,25(OH)_2_D supplementation to such patients. Vitamin D insufficiency has been associated with reduced muscle performance and loss of fast-twitch type II muscle fibers. Vitamin D may also be important for balance as measured by quantifying sway.

Several intervention studies have looked at different endpoints. Vitamin D supplementation (given daily) in deficient elderly subjects improves balance as measured by sway (99, 177). Pfeifer *et al.* (99) found that in vitamin D–deficient elders, with a mean age of 77 years and a mean baseline serum 25OHD level of 55 nmol/L, treatment with 800 IU of vitamin D_3_ per day significantly reduced body sway, when compared with the placebo group. Similarly, Cangussu *et al.* (177) found that supplementation with 1000 IU of vitamin D_3_ when compared with placebo significantly reduced body sway in 160 Brazilian women with a low mean baseline serum 25OHD level of 37.5 nmol/L. A similar conclusion was reached by Lips *et al.* (178) in older adults treated with a once-weekly dose of 8400 IU of vitamin D_3_ compared with placebo.

Based on an extensive meta-analysis, muscle (especially proximal muscle) strength may modestly improve with vitamin D supplementation of elderly subjects with serum 25OHD levels <30 nmol/L (179). Consistent with this concept, the same RCT from Brazil found that supplementation with 1000 IU of vitamin D_3_ per day for 9 months significantly reduced first fallers by nearly 50% and all falls by even more (177).

Several trials have examined the effect of vitamin D supplementation on incident fallers and fall rate. A meta-analysis of nine RCTs showed that daily supplementation of <IU of vitamin D was ineffective whereas 700 to 1000 IU significantly decreased the fall risk (180). A Cochrane review concluded that vitamin D supplementation reduced the risk of falls in institutionalized care patients (highly likely to be vitamin D deficient) [relative risk (RR), 0.63 (0.46 to 0.85)] (181). In ambulatory subjects, vitamin D supplementation did not decrease the risk of falls in a meta-analysis of all RCTs combined, but it decreased the risk of falls and fallers [RR, 0.57 (0.37 to 0.89) and RR, 0.70 (0.56 to 0.87), respectively] in subjects with a baseline serum 25OHD concentration <50 nmol/L (182). A more recent meta-analysis of RCTs found that supplementation with vitamin D reduced the fall rate only in subjects with a starting serum 25OHD concentration <75 nmol/L (183).

“There is at present no consensus regarding the potential beneficial effects of vitamin D supplementation on muscle function, balance, and risks of falls.”

In contrast, Bolland *et al.* (184) concluded from their meta-analysis that the effect estimated for vitamin D on falls lies within the futility boundary, which they defined as not altering relative risk by 15% or more. A recent *post hoc* analysis of falls in a large New Zealand (VIDA) study revealed that supplementation with 100,000 IU monthly for 3.4 years had no effect on risk of falling or fractures (104). The mean baseline serum 25OHD level of these subjects was 63 nmol/L, and less than one-third of all participants started with serum 25OHD levels <50 nmol/L.

High-dose vitamin D supplementation, however, may increase the risk of falling. This was first observed by Sanders *et al.* (102) in elderly subjects treated with a single oral dose of 500,000 IU of vitamin D_3_ or placebo once a year. The vitamin D–treated group had significantly more falls and fractures during the first 3 months after each loading dose during the 4-year treatment period compared with the placebo group. Smith *et al.* (103) found that supplementation with 300,000 IU of vitamin D_2_ annually by intramuscular injection had no effect on fall risk but increased fracture risk. In elderly women with baseline vitamin D deficiency, monthly doses of vitamin D greater than the equivalent of 800 IU/d for 1 year increased the risk of falls from 48% in the group treated with 24,000 IU monthly (equivalent to 800 IU/d) to 67% in the group treated with 60,000 IU/mo (equivalent to 2000 IU/d) and 66% in the group treated with 24,000 IU of vitamin D_3_ plus 300 μg of calcifediol per month (185). The authors concluded from a *post hoc* analysis that serum 25OHD concentrations higher than 112.5 nmol/L may be associated with an increased risk of falls. Ginde *et al.* (186) treated 107 long-term care seniors with 100,000 IU of vitamin D_3_ per month or placebo for 12 months. The monthly vitamin D decreased the rate of acute infections by 40% (primary endpoint) but doubled the rate of falls (secondary endpoint). Smith *et al.* (187) examined fall rates in 146 elderly white women (mean baseline serum 25OHD level of 38 nmol/L) treated with a full range of daily vitamin D_3_ doses (from 400 to 4800 IU) or placebo for 1 year. Falls were assessed by daily calendar and phone calls every three months. They found a U-shaped association with falls, the nadir of which occurred in the dose range of 1600 to 3200 IU/d. Fall rates in the higher doses, 4000 and 4800 IU/d, were significantly higher than those in the nadir. In contrast, among 91 African American women in the same study, there was no U-shaped association. Rather there was a progressive decline in percentage of fallers with increasing vitamin D dose, with the lowest rate occurring in the women taking 4000 to 4800 IU/d (187). This study had a small sample size for the number of dose groups but high-quality fall assessment.

### Conclusions and perspectives

There is at present no consensus regarding the potential beneficial effects of vitamin D supplementation on muscle function, balance, and risks of falls. However, overall the data seem to indicate that modest doses and daily provision of vitamin D supplementation of elderly vitamin D–deficient subjects may modestly improve muscle function, improve balance, and decrease the risks of falling. The optimal dose and dose frequency for maximal fall reduction remain to be established, as high intermittent dosing or high serum 25OHD concentrations may increase the risk of falling in white elderly subjects. Its effect in other ethnic groups remains to be explored.

## Immunity

### Innate immune system

#### Preclinical data

**Figure 4.**
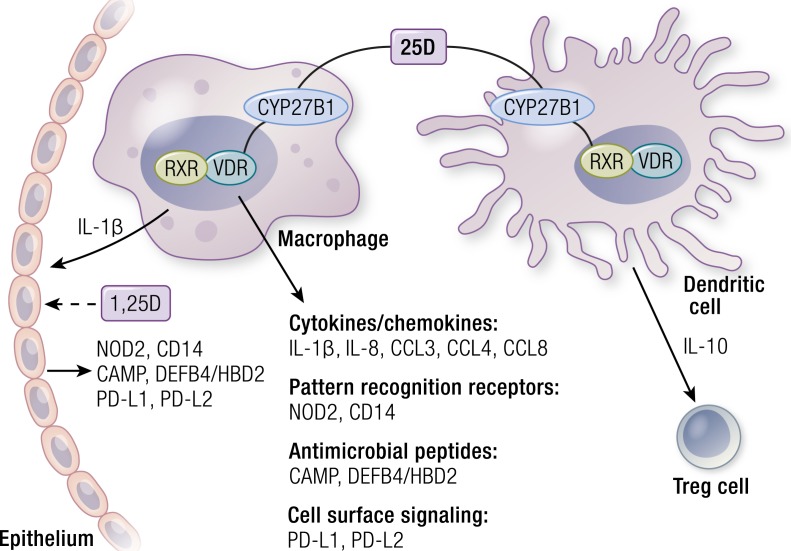
Vitamin D metabolism and signaling in innate immunity. The figure depicts intracrine production 1,25(OH)_2_D from circulating 25OHD in macrophages and DCs, as well as the effects of 1,25(OH)_2_D signaling on expression of several classes of proteins implicated in innate immune signaling. See text for details.

VDR and vitamin D metabolic enzymes are present in virtually all cells of the innate and adaptive arms of the immune system (188, 189). Importantly, there is compelling evidence that cells of the immune system produce 1,25(OH)_2_D locally and, more importantly, that expression of CYP27B1 is regulated in these cells by a network of immunoregulatory rather than calcium homeostatic inputs (189, 190). These include signaling by pattern recognition receptors, vanguards of innate immune responses to pathogen threat (190), as well as by cytokines produced by T cells of the adaptive immune system (191). *In vitro* studies of induced CYP27B1 expression in myeloid cells are consistent with clinical observations of excessive production of 1,25(OH)_2_D by macrophages in granulomatous diseases such as sarcoidosis (192). These regulatory events are important because they mirror one of the central pieces of evidence for a physiological role of vitamin D signaling in calcium homeostasis, that is, the regulation of renal CYP27B1 production by calcium regulatory hormones. Other laboratory work has provided evidence that, once activated, the VDR regulates innate immune responses upstream and downstream of pattern recognition receptor signaling by activating the transcription of several genes (summarized in Fig. 4). These encode the pattern recognition receptor nucleotide oligomerization domain protein 2 (NOD2), the Toll-like receptor cofactor CD14, antimicrobial peptides cathelicidin (CAMP, LL-37) and DEFB4/HBD2, as well as multiple cytokines, chemokines, and other signaling molecules (193–195). Notably, a combination of 1,25(OH)_2_D and IL-1β induced by 1,25(OH)_2_D in macrophages stimulated paracrine antimicrobial peptide production in epithelial cells (Fig. 3). *In vitro* studies analyzing induced antimicrobial peptide gene expression showed that conditioned media of 1,25(OH)_2_D-treated epithelial cells acquired the capacity to kill bacteria such as the lung pathogen *Pseudomonas aeruginosa* (196). Importantly, such findings were recently supported by results from a placebo-controlled, double-blind RCT, which provided evidence that vitamin D supplementation enhanced antimicrobial activity in pulmonary surface airway fluid (197).

#### Clinical data

##### Vitamin D and respiratory infections.

Numerous clinical studies have revealed associations between vitamin D deficiency and increased risk of infections, particularly of the upper respiratory tract (URT) (198). Connections between vitamin D insufficiency and infections can be traced back to the 1800s with the recognition that sunlight was beneficial for patients suffering from tuberculosis (TB). Associations between vitamin D deficiency and TB susceptibility were made in the 1980s (199, 200), as was the observation that 1,25(OH)_2_D inhibits the growth of *Mycobacterium tuberculosis* in cultured human macrophages (201). Since then, many preclinical and clinical studies have investigated the potential of vitamin D supplementation to prevent or treat TB (195, 202–206). Notably, Martineau *et al.* (203) observed in a double-blind RCT that a single dose of 100,000 IU of vitamin D_3_ enhanced antimycobacterial immunity in healthy tuberculin skin test–positive donors.

Apart from TB, multiple RCTs have provided evidence for vitamin D supplementation of deficient populations in preventing infections. A highly publicized trial published in 2010 concluded that vitamin D supplementation reduced the risk of seasonal influenza infections in Japanese children, with the effect being most pronounced in children who had not been previously supplemented (207). Other studies have provided evidence for the benefit of supplementation in populations at elevated risk for URT or ear infections due to vitamin D deficiency or a history of recurrent infections (208–210). However, results of such trials are not unanimous. For example, one study in a healthy population showed no benefit of supplementation on rates of URT infections (211), perhaps because the baseline serum 25OHD level in the population studied was 73 nmol/L. Consistent with all of the above, a recently published review and meta-analysis of individual participant data from 25 RCTs concluded that vitamin D supplementation was safe and provided modest protection (adjusted OR, 0.88) against acute URTs (212). However, subgroup analysis showed that beneficial effects were observed in patients receiving daily or weekly doses (adjusted OR, 0.81) but not in those receiving bolus doses. Moreover, in groups receiving daily or weekly doses, effects were most pronounced in patients who were vitamin D deficient (<25 nmol/L; adjusted OR, 0.30).

##### Vitamin D and inflammatory bowel disease.

Another indication where vitamin D supplementation may be of therapeutic benefit is in the treatment of patients with inflammatory bowel disease (IBD), in particular Crohn disease (CD). Although CD is often considered an autoimmune condition, it is likely driven by defects in intestinal innate immunity (213). The genetics of CD are compelling, as they reveal the importance of variations in innate immune signaling pathways, notably those controlling autophagy, in the etiology of the disease (214, 215). Vitamin D deficiency is frequent in patients with CD owing to the combination of chronic inflammation, intestinal malabsorption of vitamin D, and lifestyle. Although vitamin D deficiency has long been associated with CD, recent evidence provides a strong mechanistic basis for a role of deficiency in the pathogenesis of CD (216). For example, the gene encoding the pattern recognition receptor NOD2 (also known as IBD1) is a direct target of 1,25(OH)_2_D signaling (217). GWASs revealed that *NOD2* gene mutations disrupting its pattern recognition domain contribute strongly to CD development (218, 219).

Moreover, signaling downstream of NOD2 activates transcription of the gene encoding the antimicrobial peptide *DEB4/HBD2*, which is also a CD susceptibility locus (220) and a direct target of the VDR (196), revealing that 1,25(OH)_2_D signaling activates the extremities of the NOD2–DEFB4 innate immune pathway. The gene encoding programmed death ligand 1 (also known as B7-H1) is also a direct target of the VDR (158). Programmed death ligand 1 interacts with its receptor programmed death 1 on T cells to suppress inflammatory T cell responses in peripheral tissues, and its intestinal epithelial ablation in mice leads to inflammation via defects in innate immunity (221). These findings are intriguing in light of a recent GWAS study of 1812 individuals that linked *VDR* gene variants to alterations in the human gut microbiome (222). The study also found that *VDR* was upregulated in colonic biopsies of IBD patients, including those with CD. However, others found lower VDR levels in patients with CD or IBD, but confirmed that colitis is enhanced in the absence of the intestinal VDR and tapered down when the intestinal VDR is overexpressed (223). A role for vitamin D signaling in control of inflammation is also supported by clinical data. A large prospective cohort study of 72,719 women in the Nurses’ Health Study documented 122 cases of CD and found that for women with predicted serum 25OHD levels of 75 nmol/L the multivariate-adjusted HR for developing CD was 0.38 (95% CI, 0.15 to 0.97) when compared with those with predicted levels of <50 nmol/L. Retrospective cohort studies concluded that vitamin D deficiency was common among CD patients and was independently associated with greater disease activity (224, 225), as well as increased levels of markers of intestinal inflammation (226). Importantly, intervention trials have also provided positive results, with one finding a significant association between supplementation (*P* = 0.02), circulating serum 25OHD levels (*P* < 0.05), and disease quiescence in pediatric IBD patients (227). Others found that supplementation was inversely correlated with use of multiple fee-based services by IBD patients (228) and reduced rates of surgery in CD patients (229). Two double-blind, placebo-controlled RCTs in CD patients have been published to date. Ninety-four patients in remission were randomized to receive either placebo or 1200 IU/d for 12 months. The relapse rate was reduced from 14 of 48 in the placebo group to 6 of 46 in the treatment group (*P* < 0.06) (230). A small-scale, double-blind, placebo-controlled RCT of 27 CD patients in remission showed that 2000 IU/d of vitamin D for 3 months was sufficient to significantly increase serum 25OHD levels (231), and that supplementation enhanced circulating levels of the antimicrobial peptide LL-37 (cathelicidin, CAMP) and maintained intestinal permeability (whereas it was increased in the placebo group). Moreover, in patients whose serum 25OHD levels were at least 75 nmol/L, treatment was associated with higher quality-of-life scores (232). These findings provide strong support for conducting large-scale RCTs to examine the therapeutic efficacy of vitamin D supplementation in CD. It will be important to account for possible malabsorption in a CD patient population and to use sufficiently robust levels of supplementation.

### Vitamin D, adaptive immunity, and autoimmunity

#### Preclinical data

**Figure 5.**
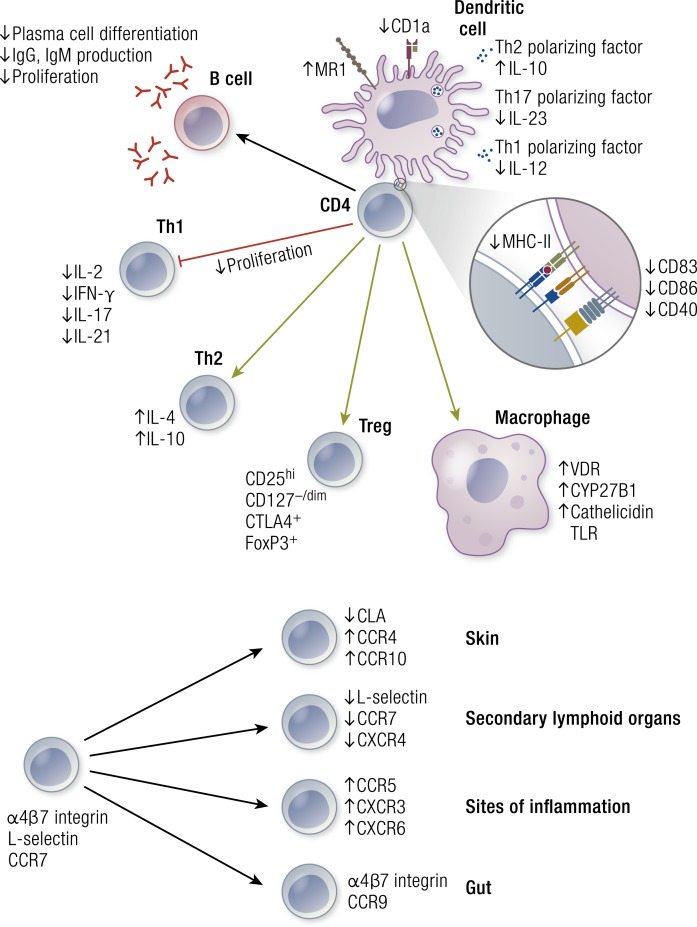
Vitamin D metabolism and signaling in the acquired immune system. In antigen-presenting cells (including DCs), 1,25(OH)_2_D_3_ inhibits the surface expression of major histocompatibility complex II (MHC-II)–complexed antigen and of costimulatory molecules, in addition to production of the cytokines IL-12 and IL-23, thereby indirectly shifting the polarization of T cells from a Th1 and Th17 phenotype toward a Th2 phenotype. Additionally, 1,25(OH)_2_D_3_ directly affects T cell responses by inhibiting the production of Th1 cytokines (IL-2 and IFN-*γ*) and Th17 cytokines (IL-17 and IL-21), as well as by stimulating Th2 cytokine production (IL-4). Moreover, 1,25(OH)_2_D_3_ favors Treg cell development via modulation of DCs and by directly targeting T cells. Finally, 1,25(OH)_2_D_3_ blocks plasma-cell differentiation, IgG and IgM production, and B cell proliferation.

We have known since the 1980s that the VDR is present in T lymphocytes and that 1,25(OH)_2_D is an inhibitor of T cell proliferation and activation (232, 233). T lymphocytes are composed of several subsets, including CD4^+^ helper T cells, cytotoxic CD8^+^ T cells, regulatory T (Treg) cells, natural killer cells, *γδ* T cells, and memory cells. The effects of vitamin D signaling on T cell subtypes have been reviewed extensively elsewhere (189, 234, 235) (Fig. 4). In general terms, vitamin D acts to suppress T cell–driven inflammation and enhance the effects of suppressive Treg cells. The communication between antigen-presenting dendritic cells (DCs) and T cells appears to be particularly important in this regard. The intracrine production of 1,25(OH)_2_D induces a more tolerogenic DC phenotype, characterized by the production of IL-10 (Fig. 5), which stimulates the production of Treg cells (189, 236). Recent studies have suggested that 1,25(OH)_2_D controls the phenotype of DCs by altering their metabolic profile (237). These anti-inflammatory effects of vitamin D signaling on DCs and T cells have stimulated extensive interest in the relationship between vitamin D status, inflammation, and autoimmunity. In autoimmune diabetes-prone NOD mice vitamin D deficiency early in life only (with normal intake later on) substantially and highly significantly increased the subsequent risk of disease in two independent studies (238, 239). In other work, dietary 1,25(OH)_2_D reduced arthritic lesions in two mouse models of autoimmune arthritis, murine Lyme arthritis, and collagen-induced arthritis (240). Very similar data have been made for experimental allergic encephalomyelitis [a mouse model for human multiple sclerosis (MS)] and IBDs. These data provide support for human studies exploring a role for vitamin D in the prevention or therapy of autoimmune diseases such as type 1 diabetes, IBD, MS, rheumatoid arthritis (RA), and systemic lupus erythematosus (188, 241).

#### Human data

Observational data have consistently confirmed an association between poor vitamin D status and all major autoimmune diseases. As detailed above, topical 1,25(OH)_2_D or its analogs have demonstrated efficacy against psoriasis, which is characterized by increased keratinocyte proliferation, but has inflammatory and autoimmune components as well. The relationship between vitamin D deficiency and MS has also attracted extensive interest. A large nested control study in US white army recruits demonstrated that a low vitamin D status (defined by serum 25OHD levels <50 nmol/L) at the time of recruitment conveyed a nearly twofold risk of later onset of MS compared with whites with a better vitamin D baseline status (242). The Finnish Maternity Cohort study reached similar conclusions (243). A similar study in patients with type 1 diabetes generated similar results (244). Several retrospective studies, albeit not optimally designed, showed that that vitamin D supplementation early in life reduced the risk of developing type 1 diabetes later in life by ∼30% (245). The dose of vitamin D required for this effect has not been clearly defined but a daily dose (up to 2000 IU) during the first year of life was most effective. This dose is markedly higher than the presently accepted or recommended dose for infants (200 to 400 IU/d). There are also well-established links between vitamin D deficiency and RA. In >29,000 women followed for 11 years, RA (152 cases in total) was inversely associated with intake of vitamin D (highest vs lowest tertile) (246). A small-scale MR study did not find a link between genetically predicted serum 25OHD and outcome parameters in patients with RA, but the study did not evaluate the risk of developing this disease (Table 2).

MR studies in Canadian, Swedish, and US cohorts have clearly shown an association between the presence of genes predisposing to lifelong lower serum 25OHD concentration and later onset of MS (Table 2). In the Canadian study (61), four SNPs known to be related to lower serum 25OHD were all independently associated with a significantly increased risk of MS. By extrapolation, 1 SD lower serum 25OHD concentration would imply a doubling of the risk of MS. In a US cohort and a Swedish cohort (total number of >7000 cases vs >14,000 controls), similar results were obtained using three SNPs related to serum 25OHD status. The overall OR was 0.85 for subjects without SNPs coding for higher serum 25OHD concentrations (62). Similar conclusions (OR, 0.72) were reached when only pediatric onset (<18 years of age) cases (n = 569) of MS were analyzed (60). The largest study (247) identified an additional synonymous variant in the coding region of CYP2R1, present in ∼5% of individuals of European origin (and much lower in Africans and Asians). This variant was associated with significantly lower serum 25OHD concentrations and a 2.2-fold increased risk of vitamin D deficiency, and in addition with a 1.4-fold OR of developing MS. These data thus strongly support the idea that lifelong lower vitamin D status due to genetically decreased serum 25OHD levels (albeit only about ≤12.5 nmol/L compared with subjects with genetically higher serum 25OHD levels) is a risk factor for autoimmune diseases such as MS. For type 1 diabetes, only one MR has been published (63) dealing with a very large number of patients (>9000). Using five SNPs predicting a lower lifetime serum 25OHD concentration, a significantly higher relative risk of type 1 diabetes of 1.07 was found in cases vs controls and of 1.10 in a family study.

Although there are preclinical and observational data to suggest that maintenance of vitamin D sufficiency should help prevent the onset of autoimmunity, current evidence for the therapeutic benefit of supplementation is inconclusive, and many studies are limited by group sizes. For example, in MS, a number of trials of vitamin D supplementation have been performed, generating contradictory results concerning parameters such as size of MRI lesions, rates of relapse, and effects on functionality (248). However, studies with type 1 diabetes [many of which used 1,25(OH)_2_D supplementation], although inconclusive, are more promising as they suggest that early intervention may preserve *β*-cell function (248). Moreover, 1,25(OH)_2_D induces a VDR-dependent transcriptional program underpinning *β*-cell survival (249). One trial concluded that a protective effect of 1,25(OH)_2_D only occurred in cases with recent (<1 year) onset of the disease (250). These findings are in line with the idea that vitamin D supplementation may contribute to prevention of type 1 diabetes (251, 252), but lose its benefit once *β*-cells are largely destroyed. RCTs are needed to confirm this observation and, if so, to define the optimal timing (*e.g.*, early in life?) and safe dosage. As type 1 diabetes as well as MS have a long silent evolution before the onset of clear clinical symptoms, such studies may require a long follow-up of subjects at increased risk for such diseases.

For other autoimmune disorders, no reliable intervention trials are yet available, but the preclinical data and observational studies on IBD are promising so that such trials deserve a high-priority score. Two large-scale studies also evaluated the preventive effects of vitamin D supplementation during pregnancy on the incidence of allergy (asthma) in their offspring (up to age 3) and the combined results showed small beneficial effects (253, 254).

### Vitamin D, atopy, asthma, and atopic dermatitis

Atopy is a predisposition to develop allergic diseases such as asthma and atopic dermatitis, and it is generally characterized by increased serum IgE concentrations. The vitamin D system has a number of effects on immune cells and cytokines implicated in atopy (234, 255). A poor vitamin D status has usually been associated with increased risk of wheezing or asthma and other aspects of atopy, although the opposite has also been reported. A Cochrane meta-analysis of two RCTs concluded that vitamin D supplementation reduced atopic exacerbations (256), but more recent data did not confirm this conclusion (257). High-dose vitamin D supplementation during pregnancy showed a small benefit in reducing wheezing in the offspring at age 3, at least when the results of two independent RCTs are combined (222, 223, 254, 255). A large MR study (dealing with >150,000 patients of all ages) showed that serum 25OHD, predicted based on four SNPs, did not influence the risk for (adult or pediatric onset) asthma, atopic dermatitis, or increased serum IgE (78). A similar conclusion was reached in another MR study on the same topic (258), and in another MR study of the GABRIEL asthma database (79). In a small-scale MR analysis of Dutch participants, genetically predicted serum 25OHD was not linked to an inflammation marker, C-reactive protein (80) (Table 2).

### Conclusions

There is broad and growing evidence that VDR and vitamin D metabolic enzymes are present in the innate and adaptive arms of the immune system, and, more significantly, that vitamin D signaling in the immune system is physiologically important and of clinical significance in patients with deficiencies. Overall, the data suggest a role of vitamin D status in sensitivity to infections and autoimmune diseases, whereas the risk of atopic diseases is less evident. Three MR studies all showed a clear significant link between genetically predicted lower serum 25OHD concentrations and the prevalence of juvenile or adult onset MS. Intervention studies to date show particular promise in CD. However, similar to RCTs in other immune-related disorders, definitive conclusions concerning therapeutic benefit are limited by study sizes. Nonetheless, the data are sufficiently compelling to merit larger-scale RCTs for CD and other immune conditions, in particular in patients with early stage type 1 diabetes or in patients at risk for developing the disease.

Thus, a link between the vitamin D endocrine system and the immune system is highly plausible, but whether vitamin D deficiency in humans has real implications for infections or autoimmune diseases has yet to be confirmed by large-scale RCTs. It will be important to perform such trials because data accumulated to date indicate that low vitamin D status enhances the risk of upper respiratory infections and that vitamin D deficiency during early life predisposes the immune system to a higher later risk of autoimmune diseases or allergy.

## Cancer

### Preclinical data

In 1979, Eisman *et al.* (259) suggested a possible association between vitamin D and cancer, when they described for the first time the presence of the VDR in a breast cancer cell line. They later showed that these receptors were present in many, but not in all, cancer cell lines and tissues, thus concluding that the VDR was not a marker for malignancy but might play a role in the pathogenesis or evolution of cancer. Additionally, CYP27B1 is expressed in many cancers, often at higher levels than in the surrounding normal tissue. Subsequently, Abe *et al.* (260) and Colston *et al.* (261) demonstrated a clear inhibition of cell proliferation of myeloma or melanoma cells, respectively. The antiproliferative effect of 1,25(OH)_2_D on cancer cells has since been confirmed in most normal and cancerous cells whereby 1,25(OH)_2_D especially inhibits cell cycle progression at the G_1_ stage. This effect usually requires nanomolar concentrations of 1,25(OH)_2_D. Therefore, many synthetic analogs have been developed (262) aiming to find a molecule with a better ratio of anticancer vs calcemic effects (*e.g.*, EB1089, inecalcitol) (262), and some of them have already been tested in humans (263). Some malignant cells may lose their sensitivity to 1,25(OH)_2_D through loss of VDR expression. However, many cancer models are wholly or partially resistant to the antiproliferative effects of 1,25(OH)_2_D in spite of retaining intact vitamin D signaling (264, 265). In contrast, overexpression of the catabolic enzyme CYP24A1 is fairly frequent in malignant cells and was even described as an oncogene in breast cancer screening (266). The inhibition of cell cycle progression can be explained by the effects of 1,25(OH)_2_D on a very large number of genes that are coherently regulated. 1,25(OH)_2_D regulates E2F transcription factor function, Rb phosphorylation, cyclin-dependent kinase activity, cMYC expression, and TGF-β and prostaglandin signaling. Moreover 1,25(OH)_2_D can inhibit angiogenesis, induce apoptosis, and decrease inflammation, invasion, and metastasis (267) (Table 3).

**Table 3.** Mechanisms of Vitamin D Tumor Suppression

Effect	Mechanism
Antiproliferative	1. Arrest of cell cycle: G_0_/G_1_ and G_1_/S
	2. Dephosphorylation of FOXO
	3. ↓ Levels of myc, fos, and jun
	4. ↓ Activity of growth factors: IGF-1, IHH, and EGF
	5. ↑ Activity of TGF-*β*
	6. ↓ Activity wnt/*β*-catenin signaling
Apoptosis	1. ↑ Expression GOS-2 and Bax, ↓ expression Bc12 and Bc1-x_L_
	2. ↑ Expression DAP-3, CFKAR, and FADD, ↓ caspases
	3. ↑ Expression PTEN
	4. ↑ Autophagy
DNA Repair	1. ↑ Clearance of cyclobutane pyrimidine dimers and pyrimidine-(6,4)-pyrimidone photoproducts (in UV-B–irradiated skin)
	2. ↓ Oxidative DNA damage by ↑ expression antioxidant enzymes
	3. ↑ Expression of DNA repair enzymes XPC and DDB2
Prostaglandin metabolism	1. ↓ COX2 expression
	2. ↓ Prostaglandin receptors
	3. ↑15-PDGH expression
Angiogenesis	1. ↓ Proliferation of endothelial cells
	2. ↓ VEGF expression
Metastasis	1. ↓ Cell migration and invasion capacity
	2. ↓ Expression of laminin and its receptors
	3. ↑ Expression of E-cadherin
	4. ↓ Expression of CEACAMI

Animal models with knockout of the VDR have been used to understand the consequences of the lack of genomic effects of 1,25(OH)_2_D on cell differentiation. Although *Vdr*-null mice do not spontaneously develop more cancers, they are more prone to a variety of malignancies, such as breast (268), colon (269), and skin (123, 270) cancer, when exposed to carcinogenic conditions, such as oncogenes, loss of antioncogenes, or exposure to carcinogens or UV-B light (17, 271). Overexpression of Cyp24a1 in mammary cells makes such animals more sensitive to breast cancer (268). A recent study in mice with genetic absence of Cyp27b1 kept on a rescue diet to maintain normal calcium homeostasis for 1 year demonstrated a higher incidence of a variety of cancers compared with wild-type mice or null mice treated with 1,25(OH)_2_D (272).

Several animal studies [Table 4 (265, 273–288)] showed some beneficial effects of less hypercalcemic vitamin D analogs on the evolution of transplanted tumors, but efficacy is nonetheless limited by potential hypercalcemia (20, 289). Indeed, a large number of studies in different experimental models have demonstrated positive effects of the active form of vitamin D against tumor growth or progression of metastases, together with histological signs of decreased cell proliferation rate, inhibited angiogenesis, and induction of cell differentiation (271). Active vitamin D metabolites and analogs have been tested in clinical trials as adjuvant treatment against cancer, although, similar to preclinical models, their efficacy is limited by the development of hypercalcemia (263) (Table 4).

**Table 4.** Selected List of Animal Studies on the Use of Vitamin Metabolites or Analogs on Cancer

Cancer	Author	Study Design	Results
Colorectal	Newmark *et al.* (273)	Western diet	Ca + D prevents
	Murillo *et al.* (274)	Chemical induced	D prevents
	Yang *et al.* (275)	APC^min^ + Western diet	Ca + D prevents
	Xu *et al.* (276)	APC^min^ + D-deficient diet	D + 1,25(OH)_2_D prevents
	Zheng *et al.* (277)	APC^min^ in VDRKO	↑ Cancers
	Huerta *et al.* (278)	APC^min^ + D-deficient diet	1,25(OH)_2_ D prevents
Breast	Lipkin and Newmark (279)	DMBA + Western diet	Ca + D prevents
	Zinser and Welsh (280)	DMBA + VDRKO	↑ Cancers
	Zinser and Welsh (281)	MMTV-neu + VDRKO	↑ Cancers
	VanWeelden *et al.* (282)	MCF-7 xenografts	EB1089 ↓ growth
	Ooi *et al.* (283)	Tumor inspections	D deficiency ↑
	El Abdaimi *et al.* (284)	Xenograft breast cancer	EB1089 ↓ growth
Prostate	Bhatia *et al.* (285)	Xenograft prostate cancer	EB1089 ↓ growth and mets
	Zheng *et al.* (286)	PC3 cells in bone	Deficiency ↑ growth
	Mordan-McCombs *et al.* (287)	LPB-tag model	1,25(OH)_2_D ↓ progression
	Krishnan *et al.* (288)	TRAMP model	1,25(OH)_2_D ↓ growth
	Chung *et al.* (265)	TRAMP model + VDRKO	↑ Angiogenesis

### Human data

Epidemiological data have described associations between solar radiation and cancer incidence and mortality, suggesting a possible role of vitamin D. The first hint for a link between cancer mortality and sun exposure was already mentioned in 1941 (290). The Garland brothers (291) expanded these studies in 1980 and reported that US populations with lower solar radiation had higher rates of mortality from colon cancer. Subsequently, many other types of cancer were described regarding similar associations to sun exposure (292), followed by dozens of meta-analyses looking for associations between low levels of serum 25OHD and higher incidence and mortality for different types of cancers, some with controversial results (293). This was observed in North American as well as with European whites and Japanese subjects (294–297), and it was concluded that a low vitamin D status was associated with a higher risk of bowel cancer, although the data for other cancers were inconsistent. This inconsistency was also noted in the 2011 Institute of Medicine report (298) and the UK consensus vitamin D position statement from a large number of UK scientific organizations, including Cancer UK (299). However, the association between a low vitamin D status and colon cancer is fairly consistent, as reported in several meta-analyses (5). The relationship between vitamin D status and prostate cancer incidence or aggressive types of prostate cancer is more complex, as different studies have found more prostate cancer in subjects with low as well as high vitamin D status. Several studies also suggest that the prognosis of patients with existing cancer may be better when they have a better vitamin D status (300).

In a systematic review, van der Rhee *et al.* (301) concluded from epidemiological data that chronic (not intermittent) sun exposure is associated with a reduced risk of colorectal, breast, and prostate cancers and non-Hodgkin’s lymphoma, but the relationship with higher levels of serum 25OHD could be demonstrated only for colorectal and breast cancer risks. Similar to data cited above, prostate cancer seems to behave differently. In Nordic men from Finland, Norway, and Sweden, the correlation between prostate cancer risk and serum 25OHD concentrations showed a U-shaped curve, increasing risk for lower as well as higher serum 25OHD concentrations (302). Although an inverse correlation between UV radiation and mortality for prostatic cancer was also described (303), a recent meta-analysis found a higher risk for prostate cancer at high levels of serum 25OHD (304), but this relationship seems to be dependent on the grade of aggressiveness of the prostatic cancer. Higher serum 25OHD levels might be associated with an increased incidence of less aggressive tumors, whereas lower levels substantially increased the risk of higher-grade (Gleason score 8 to 10) prostatic cancers (305). Overall, several meta-analyses provided a fairly consistent relationship between vitamin D status and colorectal cancer (306–309) [Table 5 (308, 310–313)]. For other tumors, such as breast, bladder, or lung tumors, the evidence is weaker or inconsistent (293). Most studies are limited to white elderly women, with scarce data for younger men and other ethnic groups such as the black population. Most of the studies concluded that high serum 25OHD levels are associated with improved cancer outcome and, less consistently, with lower cancer risk (293). Several studies also suggest that the prognosis of patients with existing cancer may be better when they have a better vitamin D status (300), and these results were confirmed using vitamin D pathway genetic variants analysis (52).

**Table 5.** Meta-Analyses of Human Epidemiologic Studies Dealing With Cancer and Vitamin D Status

Cancer		Author	No. of Studies/Analysis	Pooled RRs
Colorectal	Ma *et al.* (310)		9	0.88 (0.8–0.96) Vitamin D intake
				0.67 (0.54–0.80) 25OHD levels
	Yin *et al.* (311)		10	0.82 (0.69–0.97) 25OHD levels
Breast	Chen *et al.* (312)		11	0.91 (0.85–0.97) Vitamin D intake
			8	0.55 (0.38–0.80) 25OHD levels
				0.83 (0.79–0.87) Case control (5)
	Gandini *et al.* (308)		10	25OHD levels
				0.97 (0.92–1.03) Prospective (5)
Prostate	Gandini *et al.* (308)		11	0.99 (0.95–1.03) 25OHD levels
	Gilbert *et al.* (313)		13	1.14 (0.99–1.31) Vitamin D intake
			14	1.04 (0.99–1.10) 25OHD levels

There was inconsistency of the association between vitamin D status and breast or prostate cancer, but a consistent association with colorectal cancer.

In this regard, MR studies have, however, generated inconsistent results (Table 2). Ong *et al.* (52) found that genetically low serum 25OHD concentrations (as based on four SNPs) increased significantly the OR for ovarian carcinoma in a large (>30,000) group of whites, as well as for all types of ovarian cancer (OR, 1.27) as for high-grade cancers (OR, 1.54). Three other MR studies dealing with breast cancer or prostate cancer, however, did not find a relationship with SNPs associated with lower serum 25OHD (51, 53, 54). Additionally, two MR studies have evaluated all cancer types in relationship to four or five SNPs related to vitamin D status in a very large group of patients. Chandler *et al.* (49) studied 23,293 European women and found no significant link with genetically predicted serum 25OHD concentrations for all types of cancer, nor for specific (breast, colon, and lung) cancers or cancer deaths. Similarly, Dimitrakopoulo *et al.* (50) found no link between predicted serum 25OHD and colorectal, breast, prostate, or lung cancer in >70,000 cancer cases compared with >84,000 controls. Theodoratou *et al.* (54) also did not find a link between genetically predicted serum 25OHD and colorectal cancer in 2001 Scottish cases, although higher measured serum 25OHD was associated with lower risk of these cancers (Table 2). Dudding *et al.* (55) also did not find a link between genetically predicted serum 25OHD and oropharyngeal cancer in patients of mostly European descent living in Europe or the Americas. Finally, Sun *et al.* (56) did not find a link between genetically predicted serum 25OHD and lung cancer.

The real answer, however, about a causal role of vitamin D status on cancer has to come from RCTs. Only a few studies have so far addressed this question. Nine of 10 such RCTs [Table 6 (91, 314–321)] did not demonstrate a clear benefit of vitamin D supplementation (400 to 2000 IU/d for up to 8 years of follow-up). The large Women’s Health Initiative trial did not demonstrate a beneficial effect of combined vitamin D (400 IU/d) and calcium supplementation on cancer in general (315) or on colorectal cancer (318). Two subsequent studies of the Women’s Health Initiativedata, either excluding women who took calcium or vitamin D supplementation at baseline (319) or excluding women who took hormone replacement therapy during the study (320), revealed a better effect of vitamin D and calcium supplementation on colorectal cancer, albeit still being a nonsignificant decrease [RR, 0.81 (0.58 to 1.13 (not significant) and RR, 0.71 (0.46 to 1.09], respectively, of the risk of colorectal cancer (Table 6)]. However, excluding women on hormone replacement therapy revealed that vitamin D and calcium supplementation modestly but significantly decreased the risk of breast cancer or all invasive cancers (320) (Table 6). Trivedi *et al.* (91) randomly treated 2686 elderly individuals (2037 men and 649 women, aged 65 to 85 years) with 100,000 IU of vitamin D every 4 months or placebo for 5 years and found no differences on cancer incidence or mortality. Another extensive study dealing with colon adenoma and colon cancer did not reveal a beneficial effect of daily 1000 IU of vitamin D supplementation during 3 to 5 years, even in a high-risk population (316). Two smaller studies from Omaha, Nebraska (314, 317), provided more optimistic results on overall incidence of cancer after combined treatment with vitamin D and calcium. In the first one (314), a group of women treated with 1100 IU/d of vitamin D plus calcium for 4 years showed a significant reduction on cancer incidence compared with the placebo group, with a RR of 0.402 (CI, 0.20 to 0.82; *P* = 0.013). However, when compared with the calcium-supplemented group or the combined placebo–calcium control groups, the significant difference was lost (Table 6). Alternatively, the results of an RTC, which included 2303 elderly women from rural counties of the United States, showed that 2000 IU of vitamin D_3_ plus 1500 mg of calcium per day compared with placebo had no significant effect on cancer incidence and mortality after 4 years (*P* = 0.06) (317). However, a *post hoc* analysis excluding those who withdrew from the study, died, or developed cancer within the first year showed a significant difference between groups (*χ*^2^, 3.17% vs 4.86%; *P* = 0.046). In proportional hazards modeling, the HR was 0.65 (95% CI, 0.42 to 0.99). A limitation of this study was that most of the women included were not vitamin D deficient at baseline (serum 25OHD 82 ± 25 nmol/L); mean ± SD), although, as expected, at the end of the study the vitamin D–calcium group reached higher concentrations of serum 25OHD than did the placebo group (109 and 79 nmol/L, respectively; *P* < 0.001). Besides, all participants, including those from the placebo group, were allowed to take vitamin D supplements of up to 800 IU/d of vitamin D and up to 1500 mg/d of calcium in addition to dietary intake. Moreover, even *in situ* cancer cases were included. In line with the data described above, a meta-analysis concluded that vitamin D or vitamin D and calcium supplementation did not decrease the risk of all types of cancer (107).

**Table 6.** Vitamin D Supplementation and Prevention of Neoplasia

Outcome	Location, Trial Reference	Population	Baseline 25OHD (ng/mL)	Intervention	Duration (y)	RR (95% CI)
Total cancers	Oxford, United Kingdom, Triuvedi *et al.* (91)	2686 Men and women, age 65–85 y, living in the general population	Mean 21.4	D_3_ 100,000 IU every 4 mo vs placebo	5	1.09 (0.86–1.36)
Total cancers	Nebraska, Lappe *et al.* (314)	1179 Healthy postmenopausal women, mean age 67 y	Mean 28.8	D_3_ 1100 IU/d + Ca	4	
				Versus placebo		0.42 (0.21–0.83) (*P* = 0.013)
				Versus calcium		0.76 (0.38–1.55) (NS)
	WHI, United States, Brunner *et al.* (315)	36,282 Postmenopausal women, age 50–79 y	—	D_3_ 400 IU/d + Ca vs Ca alone	7	0.98 (0.90–1.05)
Colorectal adenomas	United States, Lappe *et al.* (316)	2259 Men and women, age 45–75 y, with at least one colorectal adenoma removed within 120 d before enrollment and no remaining polyps	Median 23.2	D_3_ 1,000 IU/d vs placebo	3–5	0.99 (0.89–1.09)

				Ca vs placebo		0.95 (0.85–1.06)
				D_3_ 1000 IU + Ca daily vs placebo		0.93 (90.80–1.08)
Total cancers (excluding skin cancers)	Nebraska, Lappe *et al.* (317)	2303 Healthy postmenopausal women, mean age 65 y	Mean 32.8	D_3_ 2,000 IU + Ca daily vs placebo	4	0.70 (0.47–1.02)
Colorectal cancer	WHI, United States, Wactawski-Wende *et al.* (318)	36,282 Postmenopausal women, age 50–79 y	Mean 19	D_3_ 400 IU/d + Ca vs Ca alone	7	1.08 (0.83–1.34) (NS)
						Comment: no effect on invasive cancers
Colorectal breast total invasive cancers	WHI, United States, Prentice *et al.* (319)	WHI 23,561 women not taking calcium or vitamin D at baseline	Mean 19	D_3_ 400 IU/d + Ca vs Ca alone	7	Colorectal:
						0.81 (0.58–1.13) (NS)
						Breast:
						0.80 (0.78–0.98) (*P* = 0.02)
						All invasive cancers:
						0.88 (0.78–0.98) (*P* = 0.03)
Colorectal cancer	WHI, United States, Dinf *et al.* (320)	8117 Postmenopausal women, age 50–79 y (excluding women on estrogen replacement therapy)	Mean 19	D_3_ 400 IU/d + Ca vs placebo	7	0.71 (0.46–1.09)
All cancers	RECORD trial, United Kingdom, Avenell *et al.* (321)	5292 Men and women, age >70 y	Median 15	D_3_ 800 IU/d vs Ca daily	3	Cancer incidence:
				Versus Ca daily		+D: 12.8%
				Versus D_3_ 800 IU + Ca daily		−D: 11.9%
				Versus placebo		HR 1.07 (NS)
						Cancer mortality:
						+ D: 5.7%
						−D: 6.7%
						HR 0.85 (NS)
Total cancers	Australia (102)	2256 Healthy postmenopausal women, age >70 y	Mean 20	D_3_ 500,000 IU once a year vs placebo	3–5	Seven cases in D group vs 10 in placebo group (NS)

RCTs with cancer as a safety or secondary endpoint with fewer than five cases of cancer per arm of the study are not included, such as in Komulainen *et al.* (389) and Prince *et al.* (390).

Abbreviations: Ca, supplemental calcium salt (citrate or carbonate); D_3_, vitamin D_3_; NS, not significant; WHI, Womens’ Health Initiative.

### Conclusions and outcome

A wealth of preclinical data support a possible role of 1,25(OH)_2_D on cell cycle progression and control of tumor growth. Total loss of vitamin D signaling also predisposes mice to the development of a variety of cancers when exposed to carcinogenic agents or genes. Poor vitamin D status is strongly linked to colon cancer. MR studies suggest a link between lifelong lower vitamin D status and risks of cancer (especially colon cancer). However, the existing intervention studies so far could not demonstrate a clear benefit of vitamin D supplementation on the incidence or evolution of major human cancers. Therefore, supplementation cannot be recommended for the sole purpose of primary or secondary prevention of cancer. Serum 25OHD levels <20 ng/mL (50 nmol/L) convey the greatest association with cancer. A few studies also suggested that serum 25OHD levels >100 nmol/L might also be associated with an increased risk for some types of cancer. However, such studies might be problematic due to the use of assays overestimating the true serum 25OHD values (322). Larger ongoing studies are needed to provide more decisive conclusions, such as the VITAL study. This is a randomized, double-blind, placebo-controlled clinical trial with a 2 × 2 factorial design with four groups: (1) active vitamin D_3_ at 2000 IU/d and active omega-3 fatty acids; (2) active vitamin D_3_ and omega-3 placebo; (3) vitamin D placebo and active omega-3; and (4) double placebo. The study population includes >25,000 subjects >50 years of age and the treatment period is 5 years. The primary endpoints are cancer and cardiovascular disease (323).

## Cardiovascular System

### Preclinical data

Several genes playing important roles in the cardiovascular system are targets of vitamin D signaling, including those encoding renin, plasminogen activator inhibitor (PAI), and thrombomodulin. *In vitro* studies demonstrate that 1,25(OH)2D has beneficial effects on endothelial, vascular smooth muscle, or cardiac muscle cells (324). *Vdr-*null as well as *Cyp27b1-*null mice develop high-renin hypertension and cardiac hypertrophy. This is in line with a negative regulation of the renin gene expression in the kidney by 1,25(OH)2D (325, 326). The cardiac hypertrophy and fibrosis probably have a dual origin: an indirect one by systemic high renin hypertension and a direct effect on cardiac muscle. Indeed, cardiac muscle-specific *Vdr*-null mice also develop cardiac hypertrophy and fibrosis that can be accelerated by increased cardiac stress (327). In line with the *in vitro* studies, *Vdr*-null mice show increased thrombogenesis and reduced fibrinolysis (325, 328, 329). *In vitro* exposure of smooth muscle cells to high concentrations of 1,25(OH)_2_D induces transdifferentiation into bone-forming–like cells with expression of several typical osteoblast-like genes, ultimately developing matrix calcifications (330).

### Observational studies in humans

Observational human studies have extensively documented an inverse relationship between vitamin D status and cardiovascular risk factors or cardiovascular events. A meta-analysis of 19 prospective studies (65,994 patients) demonstrated an inverse relationship between serum 25OHD levels (ranging from 20 to 60 nmol/L) and risk of cardiovascular disease (RR, 1.03; 95% CI, 1.00 to 1.60; per 25-nmol/L decrement in serum 25OHD) (331).

A few large studies can be summarized as follows: in the Framingham Offspring Study, vitamin D–deficient participants (serum 25OHD <37.5 nmol/L)) were more likely to have their first cardiovascular event during a mean observation period of 5.4 years in comparison with those with values ≥37.5 nmol/L (HR, 1.62; 95% CI, 1.11 to 2.36) (332). In the NHANES 2001 to 2004 study, the prevalence of coronary heart disease (angina, myocardial infarction) was more common in adults with serum 25OHD levels <50 nmol/L compared with ≥75 nmol/L (OR adjusted for age, race, and sex, 1.49; 95% CI, 1.17 to 1.91) (333, 334). Adjusting for other risk factors (body mass index (BMI), chronic kidney disease, hypertension, diabetes mellitus, smoking, and use of vitamin D supplements) attenuated the association (OR, 1.24; 95%, CI 0.95 to 1.62). The prevalence of heart failure and peripheral arterial diseases was also higher among those with serum 25OHD values <50 nmol/L (ORs, 2.10 and 1.82, respectively) with similar attenuation after adjustment for other risk factors. Several observational studies also link peripheral vascular disease with a poor vitamin D status. Indeed, an inverse dose response relationship was observed, cross-sectionally, between serum 25OHD and peripheral arterial diseases among individuals aged 40 years and older in the large US NHANES III (2001 to 2004) study (335).

### MR studies

Two independent MR studies did not find a link between genetically low serum 25OHD, predicted on the basis of SNPs related to four different genes involved in vitamin D synthesis, transport, or metabolism, and cardiovascular endpoints (69, 70) (Table 2). Similarly, a Canadian MR study did not find a link between SNPs in GC/DBP and cardiovascular diseases or stroke (71). Skaaby *et al.* (74), however, found that filaggrin-null mutations caused a 10% increased serum 25OHD concentrations due to higher efficacy of UV-B–induced vitamin D synthesis, as well as a better lipid profile. Similarly, Ooi *et al.* (72) demonstrated that SNPs in genes causing higher nonfasting remnant cholesterol concentrations also caused decreased serum 25OHD concentrations. These two MR studies suggest that the observed epidemiologic link between serum 25OHD and cardiovascular risks may be more complex and involve genes that predispose to both variations in serum 25OHD and cardiovascular risk factors (Table 2).

### Intervention studies in humans

The results of intervention studies are less convincing. In systematic reviews and meta-analyses, there was no effect of vitamin D supplementation on cardiovascular outcomes, including myocardial infarction and stroke (336–339). A meta-analysis did not show a significant effect of vitamin D supplementation on several cardiovascular risk factors such as lipids, glucose, and blood pressure. In one of the larger trials included in this meta-analyses, vitamin D supplementation did not generate a beneficial effect on cardiovascular or metabolic risks after increasing baseline serum 25OHD levels from 23 to well above 40 ng/mL (58 to 100 nmol/L) (339). Therefore, it is not unlikely that only subjects with a low vitamin D status at baseline (with precise threshold still to be defined) may benefit from such intervention.

There are several large-scale ongoing RCTs dealing with probably >100,000 subjects evaluating a wide variety of outcomes, including cardiovascular events. The data from the VIDA trial in New Zealand adults and elderly subjects were just published (340). A monthly dose of 100,000 IU of vitamin D_3_ during a mean observation period of 3.4 years did not affect cardiovascular endpoints. Whether this is due to lack of a causal relationship between vitamin D status and cardiovascular outcomes or due to other factors such as the large intermittent dose raising serum 25OHD to >125 nmol/L, to the relatively good vitamin D status at baseline (mean serum 25OHD of 63 nmol/L), is so far unknown.

#### Hypertension and vitamin D status

There is geographic and racial variation in blood pressure, with risk of hypertension increasing from south to north in the Northern hemisphere. One proposed explanation for the association with latitude is that exposure to sunlight may be protective, either because of an effect of UV-B radiation or of vitamin D (73). In observational studies of normotensive or hypertensive individuals, there is an inverse association between serum 25OHD concentration and blood pressure (341–345). However, this link is complicated by a strong negative association between BMI, a well-known risk factor for hypertension, and low serum 25OHD levels. One very large (n = 142,255 Danish subjects) MR study (73) found that a 10% increase in genetically higher serum 25OHD concentrations was associated with a 0.3 mm Hg lower diastolic and systolic blood pressure and a small but significantly lower risk of hypertension (Table 2). A 2015 meta-analysis of 46 intervention trials (evaluated at trial level or on individual patient level) did not show a benefit of vitamin D supplementation on systolic or diastolic blood pressure (345). This meta-analysis, however, did not include a trial in 283 black adults (∼50% with hypertension), showing that vitamin D supplementation significantly decreased systolic blood pressure (−1.4 mm Hg for each additional 1000 U/d of vitamin D_3_) without affecting diastolic pressure (346).

Overall, a beneficial clinical effect of vitamin D supplementation on blood pressure in unselected adults has not been demonstrated. It is unclear whether specific ethnic groups or subjects at high risk of vitamin D deficiency are more likely to benefit from vitamin D supplementation. Data on vitamin D status and supplementation (with vitamin D or active metabolites/analogs) in patients with chronic renal failure also generated no clear answer.

#### Excess vitamin D and cardiovascular risks and events

Excess vitamin D may also have major negative effects on the cardiovascular system, with ectopic (vascular) calcification, organ failure, and death as a consequence. Thus, it seems that too little but also frank excess of vitamin D may both be deleterious for cardiovascular events (325).

### Summary

There are good preclinical (biochemical, genetic, and animal data) data linking total absence of vitamin D action and adverse events in the cardiovascular system. Human observational data also link a poor vitamin D status with several cardiovascular risk factors (including all aspects of the metabolic syndrome) and cardiovascular events and even mortality. Intervention studies, however, so far are equivocal, and this may be due to lack of causality or due to poor design of the RCTs. A minimal effect of vitamin D supplementation of vitamin D–deficient subjects on systolic blood pressure is plausible (347). The causal nature of associations between vitamin D and cardiovascular disease remains uncertain. Whether the association differs across patient populations (*e.g.*, different sexes and racial/ethnic groups, chronic kidney disease, diabetes) also remains to be explored (348, 349).

## Obesity, Diabetes, and Metabolic Syndrome and Vitamin D Status

A high BMI is linked with low serum 25OHD concentration in most of the studies globally (350). Whether this is a causal link or not or whether increased fat mass causes the low vitamin D status or the other way around is not known. One hypothesis is that the fat-soluble vitamin D is more easily stored into fat cells (sequestration) before being available for further metabolism. There is, however, also a possible closed feedback loop between fat cells and vitamin D metabolism (in mice) of leptin–FGF23–Cyp27b1–serum 1,25(OH)_2_D and fat mass/leptin. Indeed, *Vdr-*null mice as well as *Cyp27b1*-null mice are leaner than controls and more resistant to diet-induced obesity by actions not completely understood but essentially due to a higher metabolic rate (351). Therefore, such mice have a lower fat mass and lower serum leptin concentrations. Second, leptin-deficient or leptin-resistant mice have higher serum levels of FGF23 because of leptin’s stimulatory effects on FGF23 production, which is known to be a major renal Cyp27b1 expression (352). Finally, to close the loop, low serum 1,25(OH)_2_D (action) decreases fat mass in mice. These observations are in clear contrast to those in humans, as a poorer vitamin D status is linked with obesity and type 2 diabetes in humans.

Vitamin D deficiency, as well as high BMI or obesity, are frequent and the (potential) consequences of both on health are overlapping. However, the precise contribution of a poor vitamin D status to obesity, its complications, and health in general are not well established.

The contribution of a poor vitamin D status to the health consequences of a high BMI or obesity is not known, but many consequences of both situations are overlapping. Indeed, all conditions known to be part of the metabolic syndrome are associated with a poor vitamin D status (353–358) in both adults and adolescents (359). One of the most consistent associations is with type 2 diabetes. For example, low serum 25OHD levels (<52.5 nmol/L)) are associated with a nearly twofold increased risk of fasting hyperglycemia or diabetes and a 1.5-fold increased risk of hypertension or hypertriglyceridemia (360). Such low 25OHD levels are also linked to a twofold increase in the overall prevalence of the metabolic syndrome (361). Longitudinal cohort studies, summarized in recent meta-analyses, have estimated an ∼40% risk reduction for incident diabetes in the highest vs the lowest category of blood serum 25OHD level (362). However, the observational nature of these studies precludes a definitive assessment of cause and effect because reverse causation or residual confounding cannot be excluded. Confounders are especially problematic in this area because blood 25OHD level is an excellent marker of good health. In relation to type 2 diabetes mellitus, higher serum 25OHD level is monotonically associated with a lower diabetes risk, suggesting no apparent threshold for benefit (362).

MR studies may help to explore the possible lifetime consequences of a low vitamin D status. Afzal *et al.* (64) concluded, based on >95,000 Danish subjects, that genetically low serum 25OHD predisposed to later type 2 diabetes. Another MR trial, however, dealing with >28,000 cases of type 2 diabetes using four known SNPs related to serum 25OHD revealed a nonsignificant OR of 1.01 (65, 295, 296). Husemoen *et al.* (67) studied ∼9000 Danish adults and found that higher predicted serum 25OHD [based on one vitamin D related SNP (DBP/GC)] was associated with a 1.6-fold increase in serum adiponectin. Such higher adipokine levels could explain a twofold lower risk for type 2 diabetes. A large MR study of 21 cohorts of European descent with obesity used four vitamin D–related SNPs but could not find a link between BMI and predicted serum 25OHD concentrations. Twelve SNPs related to higher risk for obesity, however, were found to have a significant link with higher BMI (Table 2).

Intervention studies have not demonstrated a consistent effect of vitamin D supplementation on body weight, glycemic control, or one or more aspects of the metabolic syndrome (363). The largest trial of vitamin D supplementation for prevention of type 2 diabetes is the Tromso study from Norway (511 white adults with prediabetes received 20,000 U/wk [∼2,900 U/d] vitamin D_3_ or placebo; follow-up ∼3.3 years for incident diabetes) (364). The risk of diabetes was lower in the vitamin D–supplemented group compared with placebo; however, the difference was not statistically significant (HR, 0.90; 95% CI, 0.69 to 1.18). Subgroup analyses in subjects with low baseline serum 25OHD yielded similar results. Three recent meta-analyses have evaluated the effects of vitamin D supplementation on one of three aspects of glycemic control in patients with established type 2 diabetes (365, 366). Krul-Poel *et al.* (367) evaluated 23 RCTs with a total of 1797 patients with type 2 diabetes. No significant effects were found on HbA1c, but vitamin D supplementation decreased fasting glycemia in subjects with poor baseline HbA1c. A second meta-analysis evaluated 24 RCTs, including 1528 patients with type 2 diabetes, and found a modest reduction in HbA1c (−0.3%), fasting blood glucose (-5mg/dl), or insulin resistance (all significant) following vitamin D supplementation with a mean daily dose of ∼4000 IU (or 100 μg) of vitamin D_3_.

“Vitamin D may also be important for male and female reproduction.”

They found, however, marked heterogeneity, as only 10 of 23 studies showed a positive effect. Subgroup analysis revealed some strange observations, as the supplementation therapy worked only in nonobese (BMI <25) subjects (whereas most type 2 diabetic patients are either overweight or obese) and only subjects with a normal baseline serum 25OHD (>50 nmol/L) showed an improved glycemic control, whereas no beneficial effects were seen in the vitamin D–deficient (<50 nmol/L) subjects. In a third recent meta-analysis Wu *et al.* (365) evaluated 23 RCTs dealing with >1000 patients with type 2 diabetes. Overall, mean serum HbA1c levels decreased by 0.25% overall (just significant), but the decrease in fasting blood glucose was not significant. Subgroup analysis revealed that HbA1c and fasting blood glucose, however, decreased significantly in subjects with vitamin D deficiency at baseline (<50 nmol/L) or in patients with starting BMI <30. The daily dose of vitamin D required for an improved glycemic control was ≥1000 IU/d. Both meta-analyses thus showed a modest improvement in glycemic control, but the subgroup analysis revealed very contradictory conclusions concerning which dose of vitamin D is required and which patients respond to vitamin D supplementation. The overall conclusion or interpretation of all of these data are that poor vitamin D status is frequently linked with the metabolic syndrome and type 2 diabetes. MR studies, however, did not support this hypothesis. Less than half of the existing intervention studies showed only limited beneficial effects, so that large-scale studies are needed to define its potential benefit and, possibly, its translation into clinical recommendations. The Vitamin D and Type 2 Diabetes (368) study, an ongoing trial that tests the efficacy of 4000 IU/d of vitamin D on prevention of diabetes in people at elevated risk, is expected to answer this question. While awaiting the results from such ongoing studies, it is impossible to define a serum 25OHD threshold for the prevention of metabolic events such as type 2 diabetes (369)

### Miscellaneous targets of vitamin D

As VDR is expressed in nearly all nucleated cells and its genomic activation regulates a large number of genes, it is plausible that vitamin D may have an influence on other tissues than the major target tissues described above. We herein only briefly summarize a few topics.

#### Neurologic disorders

Low serum 25OHD concentrations have been found in patients suffering from a variety of neurologic diseases such as Parkinson disease, Alzheimer’s disease, or schizophrenia (370, 371), apart from MS (discussed above in the section on immunity). MR studies found clear links between vitamin D status and MS (see above) and also with Alzheimer’s disease (58). In the latter study, dealing with 17,008 cases and 37,154 controls, 1 SD lower predicted that serum 25OHD caused a significant 1.25-fold higher risk of Alzheimer’s disease (Table 2). Two other MR trials did not find a link with schizophrenia (59) or Parkinson disease (57).

#### Reproduction

Vitamin D may also be important for male and female reproduction. Male reproduction is impaired in the Tokyo strain of VDR-null mice, and 1,25(OH)_2_D directly inhibits aromatase gene expression. This is in line with previous studies in rodents and humans demonstrating the essential role of the aromatase enzyme for male reproduction (372). A variety of data also suggest that the vitamin D endocrine system may be operative in male reproduction (373). A small-scale RCT, however, did not show improvement of sperm quality in men with subfertile sperm by vitamin D supplementation (374). A poor vitamin D status during pregnancy is associated with increased risk for the mother and child. A recent Cochrane analysis (375) and another review (376) found that vitamin D supplementation may reduce the risk of preeclampsia, increase mean birth weight, reduce the risk of small for gestational age, and reduce the risk for wheezing in the offspring at age 3 (376).

#### Liver, lung, and kidney diseases

Although the liver has a very low or undetectable level of VDR expression, hepatic stellate cells are a clear target, as selective VDR deletion causes hepatic fibrosis and a state of nonalcoholic liver disease (377).

The lung is another a possible target tissue for vitamin D action. Observational and intervention studies clearly demonstrated a possible link between poor vitamin D status and chronic obstructive lung disease [as reviewed in Ref. (378)]. Finally, vitamin D status has also been linked to a variety of other diseases such as nonalcoholic fatty liver syndrome, some eye diseases, autism, and many other diseases, as reviewed elsewhere (17, 329, 379–381).

The kidney is a major partner in vitamin D homeostasis, and renal diseases are frequently associated with lower serum 25OHD and lower 1,25(OH)_2_D concentrations. The complicated role of vitamin D in this context falls below the scope of the present review. One MR, however, strangely concluded that a lower predicted serum 25OHD was associated with a modestly but significantly higher estimated glomerular filtration rate (Table 2).

## Mortality

If low vitamin D status has such broad-ranging effects on so many (extraskeletal) tissues as described above, it would certainly increase the risk of a variety of diseases and thus ultimately increase mortality risks. No preclinical data exist on the relationship between longevity and vitamin D status. Many observational studies, as described above, associate a poor vitamin D status with nearly all major human diseases, and therefore it would not be surprising that a poor vitamin D status would be associated with increased mortality risk. Indeed, most observational studies found higher mortality rates in persons with the lowest vitamin D quartiles or quintiles in European (382) and US populations (383), with the highest mortality rates found in subjects with serum 25OHD <50 to 60 nmol/L. These very large studies also raised concerns about a U-shaped mortality curve, which suggests a slightly increased mortality rate in participants with the higher serum 25OHD levels (382, 383). However, subsequent recalibration of serum 25OHD determinations decreased the number of NHANES participants with high serum 25OHD [>100 nmol/L (40 ng/mL)], so that the apparent increased mortality in the two high serum 25OHD groups disappeared (384). A meta-analysis of eight prospective studies on >2600 participants (50 to 79 years of age) demonstrated a consistently higher overall mortality rate in participants with the lowest serum 25OHD concentrations (usually well below 50 nmol/L) (385). This observation included subjects living in different areas of the world (United States, Europe, and Japan). Surprisingly, the Leiden longevity study found that the offspring of nonagenarians (age >90 years) with at least one other nonagenarian in the family had lower (not higher as expected) serum 25OHD levels (386).

However, MR studies provide mixed evidence for a causal relationship. A small study (8417 German adults) confirmed an association between measured low serum 25OHD concentrations and mortality, but it did not find an effect of genetically lower serum 25OHD concentrations, based on only two SNPs, on mortality (343). The authors realized that this result may well be due to underpowering of the study. Another small-scale German study (3316 participants) also found no significant link between predicted serum 25OHD and mortality (85). However, a much larger Danish study (n = 95,766 and 9 to 19 years of follow-up) (84, 342) found an increased all-cause [RR, 1.3; confidence limit (CL), 1.05 to 1.61] and cancer (RR, 1.43; CL, 1.02 to 1.99) mortality with lower genetically predicted serum 25OHD. This effect was not much different from the excess mortality observed in a subgroup of this study using measured serum 25OHD concentrations (RR for overall mortality, 1.19; CL, 1.14 to 1.25).

Numerous RCTs designed to look at a variety of endpoints (mostly fractures or falls) also evaluated mortality risks as part of overall safety analysis. Three recent meta-analyses of such vitamin D supplementation studies generated similar conclusions (135, 306, 387): based on data of 22 RCTs on >30,000 participants (56 to 85 years of age with a median baseline serum 25OHD of 37.5 nmol/L) and 0.4 to 6.8 years of follow-up, vitamin D_3_ supplementation reduced all-cause mortality by 11% (387). A Cochrane analysis, including even more studies and >95,000 participants, came to a similar conclusion: vitamin D_3_ supplementation (most commonly used dosage of 800 IU/d) decreased the mortality rate by 6% [RR, 0.94 (0.91 to 0.98)], whereas vitamin D_2_ supplementation did not affect mortality, independently from calcium intake or baseline serum 25OHD (306). All-cause mortality is a hard and highly relevant endpoint and is easy to understand for the lay public. In view of the numerous large-scale ongoing intervention studies, this may well become a major endpoint to define the optimal vitamin D status and the associated recommended intake to achieve this status.

## Vitamin D and Extraskeletal Health: Summary

The vitamin D endocrine system regulates a very large number of genes in many cells and tissues not related to calcium homeostasis. This effect is seen early in the evolution of vertebrates and in mammals and humans. Thus, it is plausible that vitamin D has nonskeletal effects. A large set of observational data and some MR studies support this hypothesis. Intervention studies, however, have so far been inconsistent or generated “null effects.” The strongest data for possible extraskeletal effects of vitamin D so far deal with modest effects on muscle strength and falls, acute respiratory infections, and on mortality risks. Hopefully, a large number of ongoing (large-scale) trials will generate clearer answers.

## General Summary and Conclusion

The skeletal consequences of severe vitamin D deficiency are well established, as rickets was a disease that led to a search for an etiology for many centuries. An absence of vitamin D itself or of its active metabolites or of its receptor generates the same phenotype in animals as in humans. Vitamin D deficiency rickets can be prevented or cured by daily supplements of 200 to 400 IU of vitamin D per day. There is wide consensus that serum 25OHD levels <25 to 30 nmol/L (10 to 12 ng/mL) increase the risk of rickets. The pathogenesis of the disease is impaired intestinal calcium and phosphate absorption due to lack of their transporters, causing impaired mineralization and excess osteoid, whereas the typical hallmark of rachitic growth plates is due to hypophosphatemia-induced lack of apoptosis of hypertrophic growth plate chondrocytes in combination with a hypocalcemia-induced decrease in chondrocyte differentiation. Rickets is still endemic in some regions of the world or in some specific risk groups owing to a lack of implementation of systematic vitamin D supplementation during the first years of life.

“RCTs using vitamin D supplementation to prevent or improve possible nonskeletal diseases related to poor vitamin D status have generated mixed results.”

There is also general consensus that severe vitamin D deficiency or absence of 1,25(OH)_2_D (as in chronic renal failure) later in life can cause osteomalacia. Milder degrees of vitamin D deficiency generate secondary hyperparathyroidism in most but not all subjects of various ages and accelerates bone turnover and ultimately accelerates bone loss and the risk of fractures in the elderly.

In selected populations, RCTs with vitamin D and calcium supplementation demonstrated a decreased incidence of hip fractures and other nonvertebral fractures of ∼15%, with the effect being greater (i) in 80+ and 70 to 80 years of age persons than in persons 60 to 70 years of age, (ii) in those who are institutionalized in comparison with community living elderly, (iii) when vitamin D is combined with calcium supplementation, and (4) when compliance is >80%. Nearly all major governmental guidelines (111) confirm this policy and recommend that serum 25OHD levels <50 nmol/L should be avoided. Some expert groups (UK Scientific Advisory Committee on Nutrition) (29), however, consider this conclusion still not sufficiently validated to recommend specific supplementation beyond what is needed to prevent rickets/osteomalacia. Other experts and grassroots organizations recommend much higher intake and aim for much higher serum 25OHD concentrations based on observational data and comparison with serum 25OHD levels in individuals living in circumstances similar to those of early humans.

Because of the broad range of putative actions, there is much less consensus about the extraskeletal effects of vitamin D. Biochemical and genetic data clearly demonstrate that the vitamin D endocrine system could regulate, usually in a coherent fashion, a very large number of genes (∼3% of all genes). Evidently that includes mostly genes not related to calcium or bone homeostasis (388). Animal data largely confirm a coherent action at the cellular, tissue, or total-body level of the vitamin D endocrine system on cell proliferation, cell differentiation, the immune, muscular, cardiovascular, and other systems. These preclinical observations were made either in situations of total absence of vitamin D (action) or by exposure to supraphysiologic concentrations of 1,25(OH)_2_D or its analogs. A wealth of human cross-sectional and long-term prospective studies have linked a poor vitamin D status with a variety of human diseases as predicted on the basis of the preclinical data, including higher risk of cancer, infections, autoimmune diseases, cardiovascular and metabolic risk factors and events, and muscle dysfunction and falls. This has generated an intense interest and even irrational enthusiasm about the possible health effects of vitamin D supplements. Most governmental and scientific societies are more prudent and await further proof of causality before formulating optimal thresholds for serum 25OHD or optimal dosages beyond what is needed for skeletal effects. The proof of causality ultimately has to come from RCTs, but the recent introduction of MR studies has opened up a new strategy.

About 38 MR studies have so far looked at the possible health consequences of genetically lower serum 25OHD concentrations. Indeed, serum 25OHD concentrations have a strong genetic background as based on several twin studies. Large-scale GWASs have identified so far polymorphisms in at least four genes involved in vitamin D synthesis, transport, and metabolism in subjects of European or Asian descent that are associated with serum 25OHD levels. Such MR studies have intrinsic limitations, as the genes discovered so far only explain ∼5% of the variance of serum 25OHD and only allow the study of linear effects, whereas it is not unlikely that there is a threshold above which further increases in serum 25OHD would not affect the actions of the 1,25(OH)_2_D. Nevertheless, three MR studies (all in Europeans) so far demonstrated a clear association of genetically predicted lower serum 25OHD concentrations and the risk of MS, and one additional genetic study confirmed this association. Additionally, a large MR study in patients with type 1 diabetes confirmed a link between low serum 25OHD and the risk of this disease. As both MS and type 1 diabetes have a well-documented autoimmune origin, such data, in combination with many preclinical and observational data, make a strong case for a role of lifetime lower serum 25OHD concentrations (maybe especially early in life) and later autoimmune diseases. An MR study dealing with asthma was negative. Seven of eight MR studies dealing with a variety of cancers covering a total of >100,000 cases did not find a link between genetically lower serum 25OHD concentrations and total or organ-specific cancer incidence or mortality (Table 2). One MR study, however, found a that a lower (20 nmol/L) predicted serum 25OHD concentration (based on SNPs in the vitamin D pathway) caused a significantly increased OR of 1.27 of all types of ovarian cancer and an OR of 1.54 for high-grade ovarian cancer (52). This is unexpected, as most observational studies showed a clear link with colorectal cancer (negative in MR studies) and not with ovarian cancer. MR studies evaluating the link between vitamin D status and several aspects of the metabolic syndrome or cardiovascular events have generated complex data. Gene polymorphisms predisposing for lower serum 25OHD concentrations were not associated with higher BMI (66), higher incidence of type 2 diabetes (65), or higher incidence of cardiovascular events (71). Other MR studies, however, found a link between lower predicted serum 25OHD and adiponectin concentrations, which is a strong surrogate predictive factor for type 2 diabetes. Similarly, gene polymorphisms causing higher nonfasting cholesterol remnants are associated with lower serum 25OHD concentrations (Table 2). Hypertension was found to be associated with lower predicted serum 25OHD concentrations (73), well in line with the results of some RCTs. Mortality as studied in one large MR study was significantly linked with lower serum 25OHD concentrations predicted based on SNPs in the vitamin D pathway (84), although this was not confirmed in two smaller German studies (Table 2).

RCTs using vitamin D supplementation to prevent or improve possible nonskeletal diseases related to poor vitamin D status have generated mixed results. Null results were generated in 9 of 10 RCTs dealing with cancer (Table 6). Similarly, most RCTs dealing with aspects of the metabolic syndrome or type 2 diabetes generated “null” results. A meta-analysis of RCTs, however, demonstrated a modest effect on the risk of falls of elderly, vitamin D–deficient subjects. Vitamin D supplementation also modestly reduced the risk of upper respiratory infections or exacerbations of chronic obstructive lung disease patients. In line with an MR study mentioned above, vitamin D supplementation may have a modest blood pressure lowering effect in mildly hypertensive subjects. Based on a large number of RCTs, a modest 6% to 8% decrease in mortality risk has been observed when elderly vitamin D–deficient subjects receive modest doses of vitamin D.

Based on all of these data, the hype that vitamin D supplementation may be a cure for all major diseases of humankind is certainly not confirmed. However, several MR studies and RCTs suggest some (rather modest) beneficial effects of vitamin D supplementation for extraskeletal actions, such as muscle/falls, respiratory infections, MS, and hypertension. Local therapy with vitamin D analogs has well-documented beneficial effects on the evolution of psoriatic lesions.

According to the National Institutes of Health ClinicalTrials.gov register, ∼3000 RCTs dealing with vitamin D are still ongoing, so we may hope that within the next decade the results of these studies will further clarify the possible beneficial effects of vitamin D.

## Questions for Future Research

1. What are the precise details of the effects of UV light on skin production of vitamin D? Indeed, the vitamin D production after full-body exposure to sunlight (below or at the minimal erythema level) has been estimated to be >10,000 IU/d, whereas others found that such exposure is equivalent to a daily intake of ∼1000 IU.
2. Is measured or calculated free serum 25OHD concentration a better marker than total serum 25OHD for vitamin D status and different health outcomes?
3. What is the role of the local production of 1,25(OH)_2_D for skeletal and extraskeletal effects?
4. Which factors regulate the local activity of 1*α*-hydroxylase in different tissues?
5. High PTH, as well as low serum 25OHD levels, is associated with cardiovascular diseases. What is the contribution of each hormone on the overall effect?
6. Is the optimal vitamin D status tissue-dependent, with different thresholds for different physiological systems, and does it vary by age and race?
7. Is the use of 25OHD supplementation advantageous over vitamin D supplementation?
8. How can we explain the interindividual difference in the vitamin D status and response to supplementation?
9. What is the best approach to improve the knowledge to completely understand the action of 1,25(OH)_2_D on intestinal calcium and phosphate transport?
10. What are the effects of vitamin D deficiency during pregnancy and early life on adolescent and adult outcomes?
11. What are the mechanisms underlying tissue-specific actions and can they be best explored by selective deletion of vitamin D signaling?
12. For most other ligands of nuclear transcription factors, such as thyroid or sex steroid hormones or glucocorticoids, low and high concentrations are found to be associated with poor health outcome. Is this also true for serum 25OHD and other vitamin D metabolites?
13. There are ∼50 known metabolites of vitamin D. Do metabolites other than 25OHD and 1,25(OH)_2_D have an independent biological role?

## Abbreviations

1,25(OH)D: 1*α*,25-dihydroxyvitamin D
25OHD: 25-hydroxyvitamin D
BMD: bone mineral density
BMI: body mass index
CD: Crohn disease
CL: confidence limit
DBP: vitamin D–binding protein
DC: dendritic cell
GWAS: genome-wide association study
HR: hazard ratio
IBD: inflammatory bowel disease
MR: Mendelian randomization
MS: multiple sclerosis
NHANES: National Health and Nutrition Examination Survey
NOD2: nucleotide oligomerization domain protein 2
NMSC: nonmelanoma skin cancer
RA: rheumatoid arthritis
RCT: randomized controlled trial
RR: relative risk
SNP: single nucleotide polymorphism
TB: tuberculosis
Treg: regulatory T
URT: upper respiratory tract
VDR: vitamin D receptor
